# Rotational, vibrational, conformational and diastereomeric dimer cooling of aminoalcohols in soft supersonic expansions and the monohydrate of dimethylaminoethanol†

**DOI:** 10.1039/d5cp02019k

**Published:** 2025-07-24

**Authors:** Eaindra Lwin, Mathis J. Gölz, Nils O. B. Lüttschwager, Martin A. Suhm, Silvan Käser, Valerii Andreichev, Magalie A. Brandes, Markus Meuwly

**Affiliations:** a Institute of Physical Chemistry, University of Göttingen Tammannstr. 6 37077 Göttingen Germany msuhm@gwdg.de; b Department of Chemistry, University of Basel Klingelbergstr. 80 CH-4056 Basel Switzerland m.meuwly@unibas.ch

## Abstract

Supersonic jet expansions allow to cool molecules and to form molecular complexes over a wide range of expansion conditions, ranging from nearly effusive expansions of the pure vapour to colder expansions in carrier gases. The resulting molecular species can be probed by infrared absorption and Raman scattering. They are not in thermal equilibrium, but one can assign effective average Boltzmann temperatures for rotational, selected vibrational and in low-barrier cases even conformational degrees of freedom. If the conformational energy difference is not known, one can at least follow the evolution of competing structures with expansion conditions and from this derive relative energy sequences. For aminoethanol and its *N*-methylated variants, we explore rotational band contour analysis in OH stretching fundamentals, intensity analysis of sum and difference transitions with scaffold modes, relative intensities of isomers and the evolution of transient relative chirality to estimate the associated Boltzmann temperatures or energy sequences. The focus is on trends rather than on highly accurate numbers, which anyway depend on details like nozzle geometry or precise nozzle distance. These trends can be used for a better understanding of the vibrational spectra of other hydrogen-bonded systems. We show that the B3LYP functional is not able to describe the diastereomeric energy sequence for the dimethylaminoethanol dimer and that thermal shifts of infrared bands due to the weakening of hydrogen bonding depend strongly on the hydrogen bond strain. We also discuss high-barrier cases of conformational isomerism, which resist supersonic cooling and allow for low-temperature spectroscopy of metastable isomers. We assign the OH stretching spectra of the monohydrate of dimethylaminoethanol with an unusually strong water downshift. Finally, one of the successful machine learning-based models of the first HyDRA blind challenge is applied and improved for predicting the position of its water OH stretch wavenumber. The original model, based on computed harmonic wavenumbers for moderately strong H-bonds leads to a difference of 461 cm^−1^ whereas improvements based on VPT2 calculations for the base model reduce this to 49 cm^−1^.

## Abstract 2

Non-equilibrium thermal excitation of different degrees of freedom in hydrogen-bonded systems is explored using vibrational gas phase spectroscopy for the benefit of future computational modelling.

## Graphical abstract

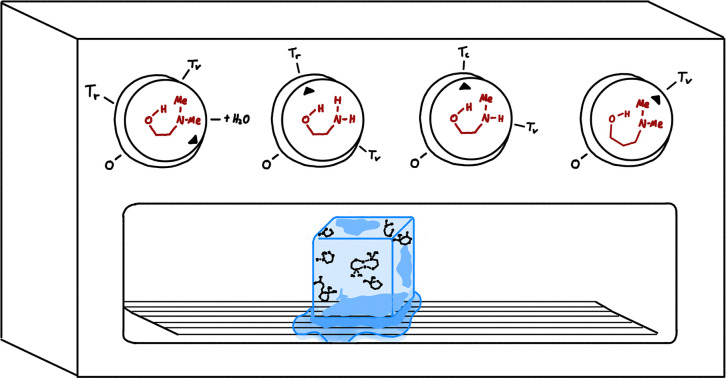

## 1 Introduction

Vibrational spectroscopy in supersonic jets^1^ is well suited to connect quantum chemical predictions with experiment,^2^ because thermal effects can be largely eliminated without having to embed the molecular systems into a perturbing cryomatrix. Once the connection has been made, it is desirable to reintroduce thermal excitation in a controlled manner. In this way, one can learn about the anharmonic effects behind spectral shifts and broadenings. Such thermal excitation effects are pronounced for hydrogen bonded systems, because hydrogen bond energies are comparable to thermal energies. The latter thus cause large spectral changes, in particular in the donor XH stretching signature. However, despite their relevance for atmospheric spectroscopy and radiation balance,^3^ there is little work addressing the evolution of vibrational spectra of hydrogen-bonded systems from cryogenic to ambient (nozzle) temperature in a systematic way.^4,5^ For more rigid molecules, direct probing of vibrational transitions^6^ and indirect probing of vibrations *via* electronic transitions^7^ to determine Boltzmann temperatures has been successful. Among others, simple refractory compounds in pure expansions^8,9^ and simple molecules in seeded jet expansions^10^ have been characterised. Buffer gas cooling has the advantage of being closer to thermodynamic equilibrium and has also been extended to larger molecules,^11^ but in most investigations using this technique, the focus is on obtaining temperatures which are as low as possible.^12^

**Fig. 1:**
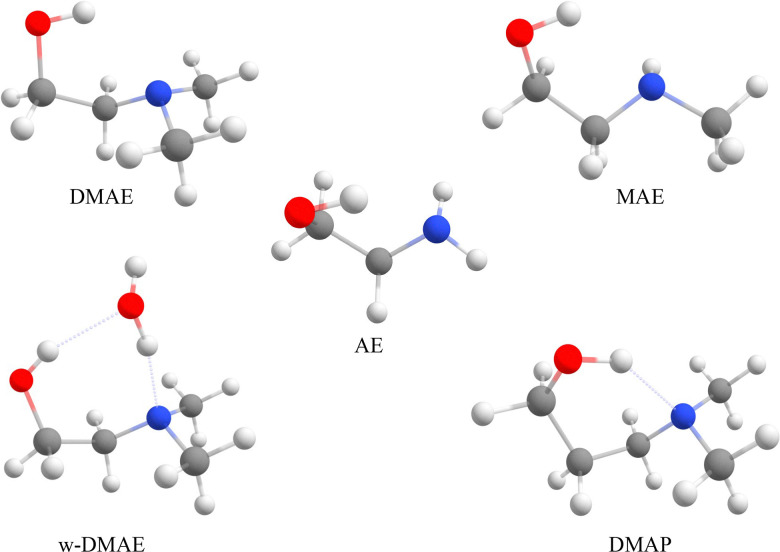
The most stable conformers of the investigated monomers (and the DMAE monohydrate) at zero-point-corrected B3LYP-D3/maTZ level: AE, MAE, DMAE, w-DMAE and DMAP.

In this study, we use the family of aminoalcohols^13^ to explore the intermediate regime between temperatures which are accessible by stationary gas phase measurements and very low temperatures achievable in seeded rare gas expansions (Fig. 1). For that purpose, we use low stagnation pressures and high analyte concentrations in the carrier gas. We also employ gas recycling techniques to reduce gas consumption and to enable long measurement series. The goal is not to derive accurate temperatures, given the non-equilibrium nature of supersonic jets and the need to average over different nozzle distances to enhance the sensitivity of our main tool, direct absorption FTIR spectroscopy. Instead, systematic temperature trends for different degrees of freedom as a function of stagnation pressure and analyte concentration will be explored. The degrees of freedom include molecular rotation, low frequency vibrations and conformational interconversion across barriers. The main focus is on internally hydrogen-bonded molecules, but conformational relaxation in molecular dimers is also explored and conformer assignments are proposed.

Aminoalcohols and their complexes have previously been studied not only in solution^14^ and in the gas phase^13^ but also in supersonic jet expansions.^15–17^ Permanently chiral derivatives of aminoethanol are easily accessible from chiral amino acids, but here we focus on the transient axial chirality of achiral aminoalcohols with respect to the OCCN dihedral angle.^18^ Aminoalcohols can also serve as proxies to amine hydrate complexes^19^ with the advantage that they survive higher rotational temperatures because of the chemical connectivity of the hydrogen bond donor (OH) and acceptor (N).

We explore three different carrier gases for the aminoalcohol analytes. Helium achieves moderate cooling due to its small mass and momentum transfer, but there is less analyte aggregation and no carrier gas condensation to compete with the initial cooling. Neon achieves faster and stronger cooling but it promotes analyte aggregation which may limit the cooling effect. N_2_ also shows fast cooling, but it can easily aggregate on the analyte clusters. The resulting N_2_ nanomatrices modify the analyte spectra due to the matrix shift and they can warm up the expansion downstream the nozzle exit.

The investigation of aminoalcohols at different effective temperatures sets the stage for a spectroscopic characterisation of the complex of dimethylaminoethanol^15^ with a single water molecule. The water is found to predominantly insert into the intramolecular hydrogen bond and its hydrogen-bonded OH stretching vibration is shown to be strongly downshifted. Due to the double methylation, no mode mixing with NH stretching vibrations is possible. The strongly downshifted experimental OH stretching wavenumber serves as a range-extending benchmark for theoretical models aiming at the prediction of such downshifts.^20^

## 2 Experimental details

The experimental FTIR spectra recorded for this work were obtained by pulsed expansion of gas mixtures at variable pressures through a homebuilt adjustable 700 mm slit nozzle into a vacuum buffer, synchronised to interferometer scans in the OH stretching range. To reduce the consumption of carrier gases and analytes, the gas mixtures are recompressed and reused hundreds of times. This gas recycling concept^21^ was modified to allow for the expansion of gas mixtures below 300 hPa stagnation pressure by a modification of the final compression stage. Instead of a screw pump (Cobra, Busch) which required at least 300 hPa gas pressure at the outlet, more flexible multi-stage roots pumps (ACP 40 CP, Adixen/Pfeiffer or Ecodry 65, Leybold) were explored. For the Raman spectra, the gas recycling concept was adapted to continuous operation through a small slit nozzle, again using an Ecodry 65 multi-stage roots pump.^19,22^ More details are provided in the ESI,† Section S1.2, with the FTIR setup Fig. S1, also about the investigated compounds in ESI,† Table S1 and all measured spectral information are described in Tables S2–S4 (ESI†).

Band integration was carried out using a numerical approach which includes the noise characteristics of the spectrometer.^23^ Further information about the integration method is provided in the ESI,† Section S3.1.

## 3 Modeling and nomenclature

2-Aminoethanol and 3-aminopropanol are abbreviated AE and AP, respectively. DM (M) is added to indicate double (single) methylation of the amino group (Fig. 1). When fundamental OH stretching vibrations of monomeric aminoalcohols (M) are accompanied by sum and difference transitions with low frequency intramolecular modes (L), these are denoted OH+L and OH−L, respectively. In homodimers (D) of aminoalcohols, the hydrogen bond topology is distinguished. Insertion of one OH group into the internal hydrogen bond of the other is denoted i and mutual OH hydrogen bonding to the nitrogen atom of the other monomer is denoted m. The relative sign of the transient chirality of the two monomers in the dimers is denoted hom (if it is the same) and het (if it is opposite). For further nomenclature details, see ESI,† Section S5.1 with Fig. S18 and S19.

Previous computational studies on these internally hydrogen-bonded species include ref. 13 and 24–26. Here, we use the ORCA program package^27^ for B3LYP-D3 optimisations and harmonic wavenumber calculations at triple and quadruple zeta level, which are extended to B2PLYP optimisations and DLPNO-CCSD(T) electronic energy corrections, where needed (for details, see ESI,† Section S4). Spectral assignments supported by computations are discussed in ESI,† Section S5.

## 4 Individual Boltzmann temperature approaches

In the absence of rotational line resolution, accurate rotational temperatures can in principle still be determined by rotational band contour simulation of the IR spectra. However, this requires the knowledge of rotational constants, rovibrational couplings, vibrational hot band contributions and some estimate of the intramolecular vibrational energy redistribution. Here, we use a simplified and generalizable correlation approach which assumes that the rotational contour roughly scales with the square root of the absolute temperature, as in the case of PR-branch separations in diatomic molecules.^28^ The room temperature profile is used for calibration. Because we are dealing with internally hydrogen-bonded molecules, where vibrational hot bands tend to distort the high frequency side of the band contour of an OH stretching transition, we simply use the lower HWHM (half width at half maximum of the P-branch) of the OH stretching band to estimate the rotational temperature. Low temperatures will be uncertain (upper bounds) due to the unknown homogeneous bandwidth at 0 K. By assuming that it is zero, instrumentally limited or given by a high stagnation pressure experiment, one can obtain a realistic uncertainty estimate. In contrast, the uncertainty of the reference room temperature is negligible compared to other assumptions such as a homogeneous temperature over the IR-probed jet expansion cross section. Raman spectroscopy is less suitable for a rotational temperature estimate in the present cases due to the typical weakness of O- and S-branches.

Vibrational band profiles of internally hydrogen-bonded systems have been analysed before in terms of sum, difference and hot bands (see Fig. S16 in the ESI†) in warm gas phase spectra.^29^ The hot bands broaden and distort the fundamental transition, whereas selected low frequency modes with anharmonic coupling to the fundamental give rise to side bands. Low-frequency side bands due to difference transitions are sensitive to the vibrational temperature, because they rely on excited state population of the low frequency mode. In the thermalised gas phase, the vibrational temperature is known and helps to analyse the intensity pattern. This is not the case for jet expansions, but in combination with thermalised spectra it provides estimates for the vibrational temperature of a specific low frequency mode. Because low frequency modes cool rather efficiently, such an approach only works for relatively mild expansions, where the difference transitions remain detectable due to residual population of the corresponding excited low frequency states. Each low frequency state may have a different cooling rate. To obtain an estimate of the uncertainty in the derived vibrational temperatures, we compare two approaches. In the more sensitive one, the intensity ratio between the difference band and the fundamental transition is followed as a function of stagnation pressure, with calibration by the room temperature gas phase measurement. This may suffer from unbalanced hot contributions to the fundamental, beyond those of the low frequency vibration of interest. By instead following the intensity ratio between the difference transition and the weaker sum transition, the hot contributions may cancel out to a higher degree, but the sensitivity is largely reduced because sum bands (whose intensity enters the denominator) can be very weak. For more details, see Section S3 of the ESI.†

Conformational temperatures have been determined before by freezing the gas phase distribution in a matrix.^30^ Here, we attempt to follow conformational relaxation with progressive cooling in the jet expansion.^31^ This only works if the barriers to be overcome are low enough or if they only arise during the interaction of molecules in molecular dimers. If the energy difference between conformations is known or can be estimated, an effective freezing temperature can be derived, at which interconversion comes to a halt. Otherwise, one may still be able to energetically order conformations by following the interconversion.

## 5 Results

### 5.1 Rotational temperatures *T*_r_

**Fig. 2:**
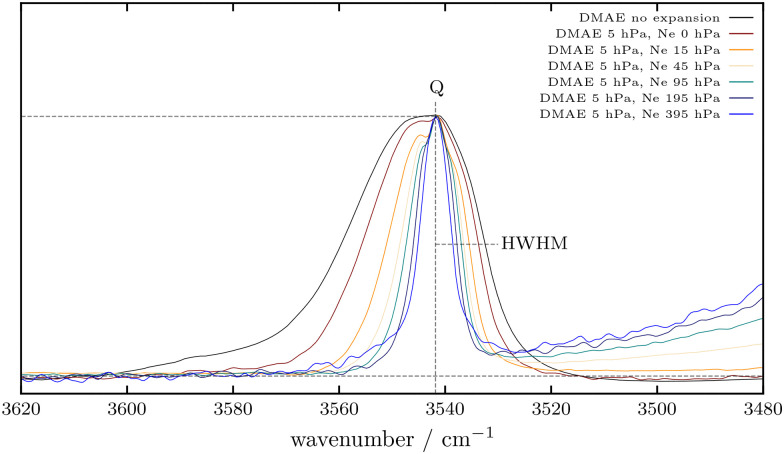
Evolution of the OH stretching fundamental of 5 hPa DMAE with the addition of increasing amounts of carrier gas to the expansion, scaled to the same peak height. Also shown is the stagnant gas phase spectrum which serves as a temperature reference. The low-frequency half-width at half-maximum is used for a rotational temperature estimate.

Fig. 2 shows spectra of 5 hPa DMAE (2-dimethylaminoethanol) in the OH stretching fundamental range, scaled to the same peak height. Increasing amounts of Ne carrier gas lead to a narrowing of the band profile due to rotational cooling, but also to increasing contributions from clustering on the low frequency side. These extend slightly to the high frequency side, where vibrational hot bands also contribute notably due to the weakening of the intramolecular hydrogen bond. Corresponding spectral series in He, at lower concentration and for AE are shown in the ESI,† Fig. S2–S8.

Effective rotational temperatures *T*_r_ can be estimated from the width of the band profile (on the low frequency side which is comparatively free from vibrational hot band congestion) relative to the width in the gas phase at the stagnation temperature *T*_s_, assuming an approximate square root dependence of the width on the temperature. For a systematic comparison and trend analysis, we use a range of simplifications and limiting cases.

For an upper limit of the rotational temperature, we imply that the OH stretching band has negligible width at 0 K (no instrumental or IVR broadening). All the broadening observed is attributed to thermal excitation. We assume that the temperature extracted at 400 hPa stagnation pressure is the lowest achievable (*T*_∞_).

For a lower limit of the rotational temperature, we determine the HWHM for the coldest available 400 hPa spectrum of the compound (see ESI†) and attribute it entirely to spectral resolution and IVR. We subtract this half-width from the experimental half-width at lower stagnation pressures and in the gas phase and assume that the arithmetic difference reflects the thermal contribution (which is an approximation for non-Lorentzian band profiles). The resulting temperatures for the upper and lower limits are averaged to *T*_r_.

We then plot the function *Φ* = ln(*T*_r_/*T*_∞_)/ln(*T*_s_/*T*_∞_) which runs from 1 at *T*_r_ = *T*_s_ (no expansion, formal stagnation pressure 0) to 0 at infinite stagnation pressure (*T*_r_ = *T*_∞_). It has positive curvature and becomes steeper for efficient rotational cooling. We find that *Φ*(*p*_s_) can be fitted reasonably well by a uniform exponential function (exp(−(*p*_s_/hPa)^3/4^/*c*) where 3/4 was obtained by trial and error as a compromise exponent for the available data set. *c* is a measure of the relative cooling efficiency and depends on the carrier gas and analyte partial pressure. The smaller *c* is, the faster the limiting temperature *T*_∞_ is approached with increasing *p*_s_.

**Fig. 3:**
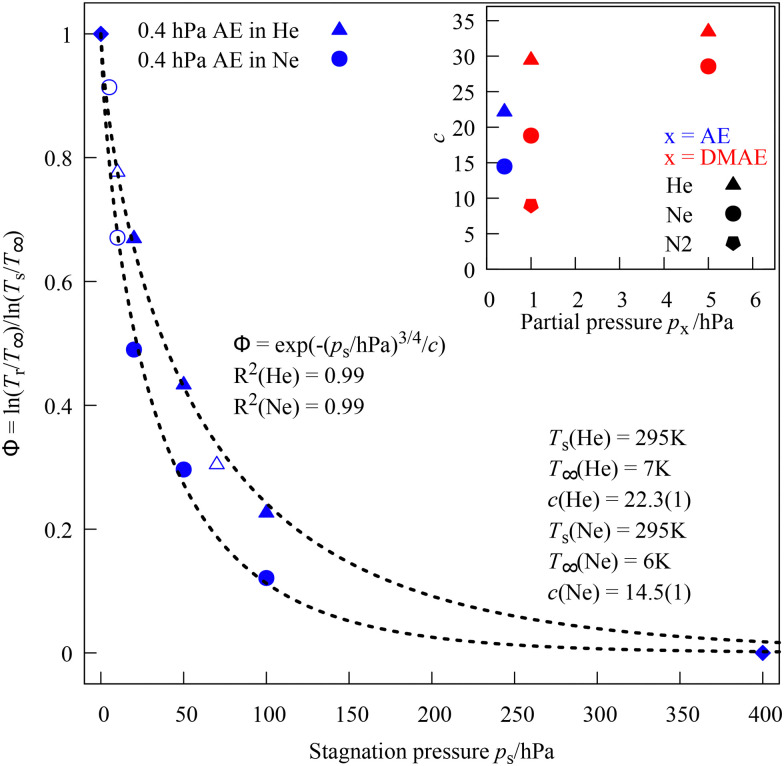
Interpolation function *Φ*(*T*_r_) between stagnating and expanding AE at 0.4 hPa mixed with He and Ne up to 400 hPa stagnation pressure (diamonds). The smaller the fitted *c* parameter and the heavier the carrier gas, the faster the rotation cools with increasing stagnation pressure. Filled symbols are included in the fit, empty symbols are test data. The insert shows fitted *c* parameters as a function of partial pressure for different aminoalcohols and carrier gases. See ESI† for details.

Fig. 3 shows a pair of example fits for the expansion of 0.4 hPa AE in He and Ne. One can see that at this high dilution, the cooling proceeds substantially faster for Ne (*c* = 15 instead of 22) but only reaches a slightly lower limiting temperature *T*_∞_ (6 instead of 7 K) in this case. Filled symbols represent common data points which are used for the fitting, whereas empty symbols represent additional stagnation pressures which were not probed for all species and carrier gases (see ESI,† Fig. S9–S15, for additional *Φ* plots).

The compound, carrier gas and partial pressure dependence of the fitting parameter *c* is shown as an insert in Fig. 3. It may be used to anticipate the approximate rotational cooling of other compounds in the slit jet expansion. More work needs to be done to find out whether *c* is reasonably transferable between different compounds and whether the partial pressure or the degree of saturation of the vapour is a more robust control parameter.

**Fig. 4:**
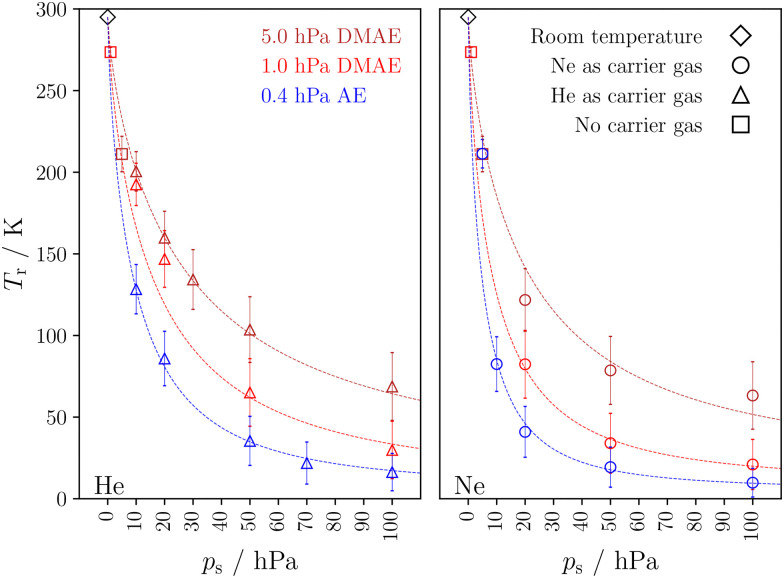
Estimated rotational temperature *T*_r_ in K as a function of stagnation pressure for He (left) and Ne (right) expansions together with the fitted functions and uncertainties arising from the unknown homogeneous band profile. See ESI,† Tables S5 and S6 for detailed information.

Fig. 4 shows actual rotational temperatures *T*_r_ (with uncertainties from the limiting assumptions) up to a stagnation pressure of 100 hPa He or Ne together with the fits obtained for *Φ*. The curves do not cross, which means that the initial cooling efficiency correlates with the cooling extent at 100 hPa.

**Fig. 5:**
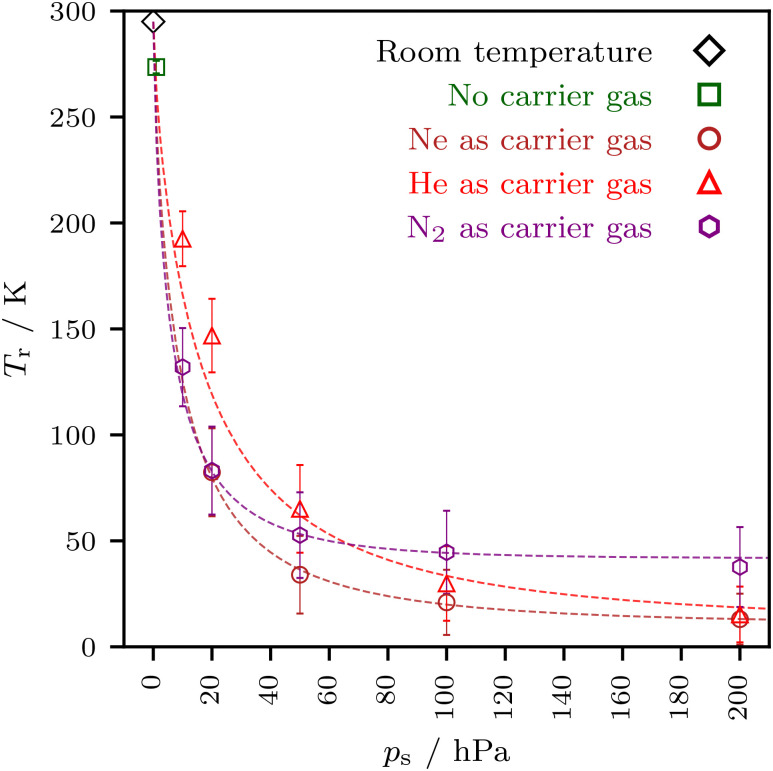
Comparison of DMAE rotational temperatures *T*_r_ as a function of stagnation pressure for noble gases and for N_2_. N_2_ is found to cool as efficiently as Ne at low stagnation pressure but less well than He at higher stagnation pressure.

Fig. 5 compares rotational temperatures obtained for N_2_ with those of the noble gases up to 200 hPa. Initially, the cooling efficiency of N_2_ is comparable to that of Ne, but with increasing stagnation pressure, presumably due to the condensation of N_2_ on the analyte and also due to enhanced analyte self-aggregation, the apparent cooling saturates and the curve crosses even the one of He.

### 5.2 Vibrational temperatures *T*_v_ and thermal shifts

**Fig. 6:**
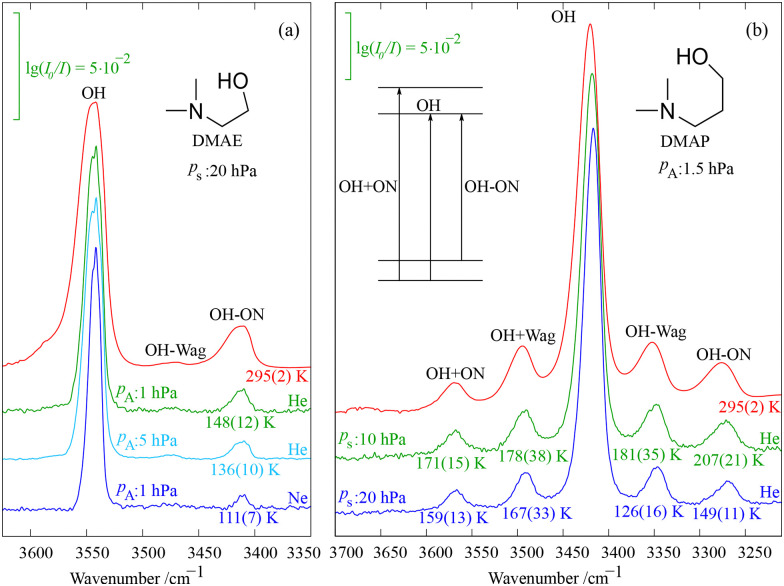
Estimated vibrational temperature *T*_v_ in K of low frequency hydrogen bond stretching (ON) and dimethylamino wagging (Wag) modes in soft supersonic jet expansions of partial pressure *p*_A_ as a function of carrier gas for DMAE (a) and as a function of stagnation pressure *p*_s_ for DMAP (b) derived from reference room temperature spectra (red). See text and ESI,† Section S3, in addition to Tables S10–S12 for further explanations.

For soft, diluted expansions with pronounced coupling of the OH stretching fundamental to a low-frequency mode, the vibrational temperature *T*_v_ of this low-frequency mode can be estimated from the intensity ratio of the difference transition (starting with low-frequency excitation and ending in the pure OH stretching state) and the OH stretching fundamental. Comparison to the room temperature spectrum, where the vibrational temperature is known, provides the degree of depopulation of the low-frequency fundamental. This is illustrated in Fig. 6a for the ON (hydrogen bond) stretching vibration of DMAE at a stagnation pressure of 20 hPa, after scaling of the spectra to the same peak OH stretching intensity. If 19 hPa are due to the He carrier gas (and 1 hPa due to DMAE), the difference band is strongest. When 5 hPa DMAE are expanded in 15 hPa He, it is weaker because DMAE–DMAE collisions are more efficient in cooling the expansion, as long as they do not lead to clusters and therefore do not generate additional aggregation energy. When He is completely replaced by Ne, the difference band shrinks even more in intensity, because the heavier Ne cools the low frequency modes better than the lighter He. Assuming that hot band contributions adding to the cold OH stretching and difference transitions cancel to some extent, the vibrational temperatures given in the figure can be derived from integrals of the corresponding bands, in relation to the gas phase spectrum. Tentatively, we assign an uncertainty of ±5% to all band integrals (including uncertainties due to hot band contributions) in the jet spectra to estimate uncertainties in *T*_v_, neglecting minor individual contributions to the uncertainty such as the temperature of the reference spectrum or spectral noise. Note that there is a second difference band at about half the frequency, matching a wagging mode of the dimethylamino group (Wag). Due to its weaker coupling to the OH stretching vibration, it remains weak and its vibrational temperature cannot be estimated. If sum bands, where OH stretching and low-frequency excitation are combined, were strong enough, they could provide an independent estimate for the vibrational temperature. This is not the case for DMAE, also because of spectral overlap with rovibrational water impurity transitions.

Fig. 6b shows the more favourable case of DMAP, where two pairs of sum and difference bands of considerable intensity flank the OH stretching transition. They are again due to ON stretching (higher frequency) and dimethylamino wagging (lower frequency) modes and now allow for two independent *T*_v_ estimates for a stagnation pressure of 20 hPa, in combination with the known temperature of the gas phase spectrum. Reduction of the stagnation pressure to 10 hPa provides a spectrum intermediate between the gas phase and the 20 hPa expansion. From an analysis of the relative integrals, approximate vibrational temperatures can be derived for both expansion conditions and are provided underneath the sum (for the sum-based method) and difference (for the fundamental-based method) bands. Their error bars estimated from the considerable integration uncertainty (again assumed to be ±5% for the jet spectra relative to the gas phase spectrum) are listed under the difference bands (derived from the fundamental intensity) and under the sum bands (derived from sum band intensity). The spectral overlap of the thermally broadened bands also can introduce systematic errors, in particular for the sum bands, which are overlapped by the high-frequency tail of the fundamental transition. A measure for this overlap is the intensity ratio between a sum band maximum and its neighbouring spectral minimum at lower wavenumber (towards the fundamental). In a cold spectrum, this minimum would have zero intensity, but in the warm spectra it remains positive. Therefore, we correct sum band integrals (jet and gas phase) by one half of that ratio before the uncertainty analysis (see ESI,† Section S3.1 and Table S9). The resulting vibrational temperatures for the two approaches differ, but largely fall within error bars. It is difficult to say which of these semiquantitative approaches is more accurate, because of the hot band contributions to the band profiles. Together, they may span the actual values. Perhaps the fundamental band method is more accurate, because it suggests that the lower frequency mode (Wag) cools more efficiently than the higher frequency (ON) mode, in line with the energy gap law.^7^ It also indicates that the temperature drops with increasing stagnation pressure. Trends as a function of stagnation pressure are more reliable than absolute values, because the strongly overlapped room temperature reference is avoided in this case, but even these trends are only weakly significant. In the future, further examples will have to show whether our coarse-grained approach to vibrational temperature leads to systematic trends.

One effect of the population of low-frequency modes is their influence on the peak position of the high frequency OH stretching vibration. For a free OH group, increasing temperature usually results in broadening, whereas for hydrogen-bonded OH groups, the broadening is accompanied by a shift towards the free OH signal. While the latter position is somewhat undefined in an internally hydrogen-bonded system, it is worth comparing a range of systems from this work and the literature, to search for systematic behaviour. For aminoethanol and aminopropanol, the OH stretching wavenumber of conformations without intramolecular hydrogen bond have been located at 3679 and 3675 cm^−1^, respectively.^13^ By Raman spectroscopy, the aminoethanol value was recently^32^ conformationally resolved to 3670/3691 cm^−1^. For DMAP, essentially the same value of 3675 cm^−1^ as for aminopropanol has been reported,^33^ suggesting a robust free OH transition (close to that of *trans* ethanol^34^). This may be compared to the hydrogen-bonded values of 3542 cm^−1^ (DMAE, both jet and room temperature gas phase) as well as 3412 cm^−1^ (DMAP, jet) and 3419 cm^−1^ (DMAP, gas phase^33^). Thus, the thermal effect for the DMAE band position is negligible, while that for DMAP amounts to ≈3% of the shift from the free OH to the hydrogen-bonded OH. This increased thermal shift may be explained by the more flexible character of the backbone surrounding the hydrogen bond in DMAP. Indeed, a completely unconstrained counterpart would be the noncovalent complex between methanol and trimethylamine, for which a thermal blueshift at room temperature reverting more than 10% of the hydrogen bond shift was recently demonstrated in jet spectra.^35^ It will be interesting to check this correlation between thermal blueshift and flexibility for other internally hydrogen bonded molecules and complexes under jet cooling conditions.^5,36–38^

### 5.3 Conformational temperatures *T*_c_ and energy sequences

#### 5.3.1 The difficult case of MAE

2-(Methylamino)ethanol (MAE), like its homolog prolinol,^39^ shows two similarly populated conformations which can be classified as *g* (*gauche*) and *t* (*trans*) with respect to the CNCC(O) backbone. These two are very close in energy and separated by substantial interconversion barriers, such that detection of a conformational temperature change in jet experiments is challenging, although the two conformations have well separated OH stretching signals at low enough temperature and can thus be discriminated.^15^ A room temperature microwave spectroscopy study derived an energy advantage of the *t* conformation by about 2 kJ mol^−1^.^25,40^ DFT calculations including harmonic zero-point energy correction and DLPNO-CCSD(T) energy corrections provide a robust picture, with an electronic energy advantage for *t* of less than 0.5 kJ mol^−1^ and a zero-point energy correction of the same sign and similar size. Thermal corrections are even smaller, such that the total computed energy advantage of *t* is less than 1 kJ mol^−1^ (ESI,† Tables S21, S22 and Fig. S25). The harmonic spectral prediction is also robust, with *t* expected 20–25 cm^−1^ higher in wavenumber than *g*. This matches the experimental splitting of 27 cm^−1^ and the slightly higher intensity of the *t* signal (for comparable IR band strengths). All attempts to substantially relax the *g* conformer by increasing the stagnation pressure and by switching from Ne to N_2_ failed (ESI,† Fig. S26). If anything, the *g* signal slightly increases, but that could be due to preferential clustering of the *t* conformer with the carrier gas or with itself. Therefore, we conclude that the interconversion barrier is too high to be overcome and the driving force too low in carrier gas collisions, in contrast to lower-barrier examples.^41^

#### 5.3.2 Selective relaxation in DMAE dimers

**Fig. 7:**
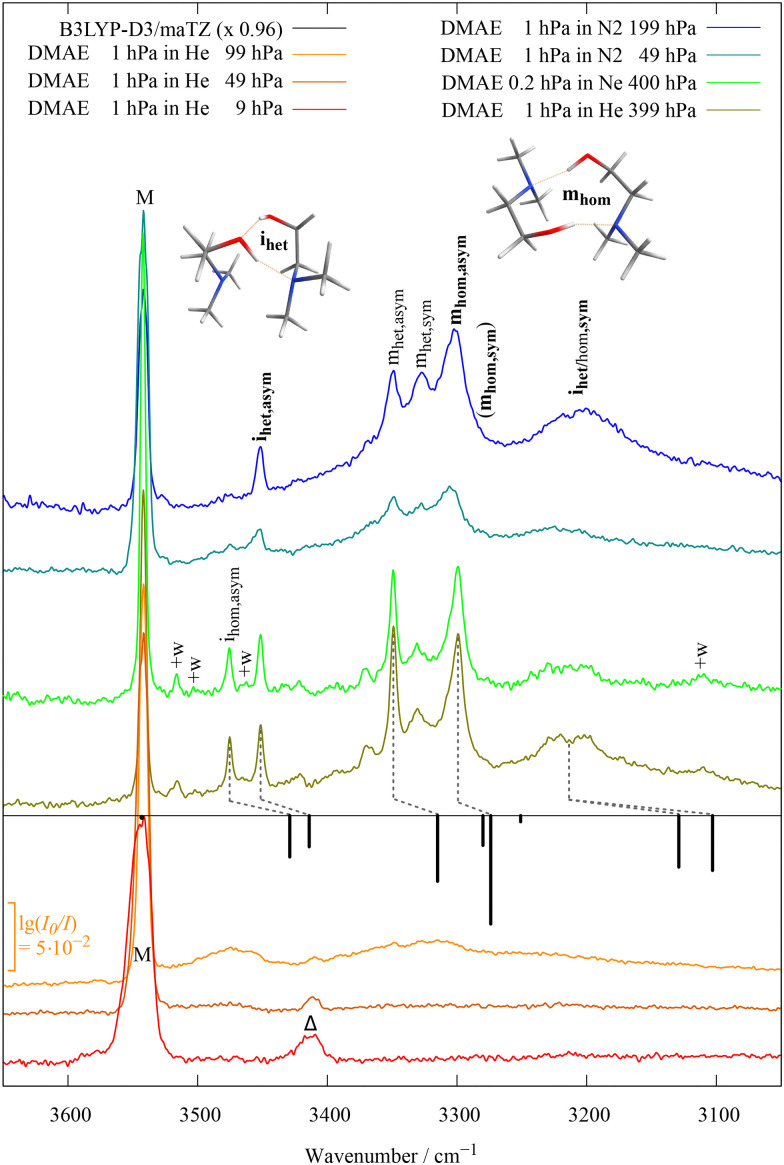
IR spectra of dimethylaminoethanol (DMAE) for different expansion conditions with He (bottom), Ne (center) and N_2_ (top) as carrier gases and comparison with harmonic prediction for monomer and dimers (black). Assignments in bold refer to the more stable conformations of a given hydrogen bond topology. See text and ESI† for details.

Dimers of aminoalcohols are more suitable for relaxation experiments, because some of the barriers separating isomers only emerge upon complexation and others can be overcome by the energy released upon complexation. To illustrate the power and limitations of carrier gas and stagnation pressure changes on conformational preferences in such dimers, Fig. 7 shows the OH stretching region of dimethylaminoethanol (DMAE) expansions, normalised to the same DMAE density for all spectral traces based on the CH intensity. To the left, there is the strong and relatively sharp monomer OH stretching signal (M) which was discussed in the context of rotational temperature. The lowest trace (red, a very soft expansion in helium) still shows a difference band with a soft intramolecular mode (marked Δ). As the population of that state is depleted at higher stagnation pressures (upper traces), it disappears. Instead, a range of cluster bands emerges. Four small ones (marked +w) are due to the monohydrates of DMAE and will be discussed later (see also ESI,† Section S6). The upper three +w bands are close to strong and sharp transitions which are due to homochiral and heterochiral pairings of two DMAE units featuring transient torsional chirality with respect to the NCCO scaffold, as elaborated in the ESI† (Section S5.2). It is remarkable that the dimer spectrum is so much more complex than the related case of aminoethanol dimers.^15,17^ Apparently, the additional methyl groups raise the interconversion barriers between the conformational manifold in such a way that metastable structures get trapped behind them. This invites variation of the expansion conditions such as stagnation pressure and carrier gas. For soft expansions, the two homodimer signals are still missing or thermally blurred, but as soon as the He stagnation pressure exceeds 100 hPa, they become well separated, with a slight intensity advantage for the more downshifted one. This advantage does not improve substantially when the carrier gas is switched from He to Ne, but the less downshifted peak is strongly depleted when N_2_ is used as a carrier gas. Comparison to simple scaled harmonic quantum chemical calculations (inverted black sticks) suggests that the disfavoured structure is due to homochiral pairing in such a way that one OH group inserts into the monomeric OH⋯N hydrogen bond of the other (we call that type of dimer geometry i(nserted),^16,17^ the N atom of the inserting monomer is not bonding to the acceptor monomer). A more complex group of bands arises further downshifted. It comprises two band patterns near 3300 and 3200 cm^−1^, where only the former gives rise to spectral structure and shall be discussed in more detail. It consists of two strong and two to three weaker features superimposing a broader envelope. The two strong features show a similar carrier gas pressure and composition evolution as the inserted pair, but the higher-wavenumber peak can only be partially depleted in favour of the lower-wavenumber peak. This is even true for N_2_ at the highest stagnation pressure, where it is likely that N_2_ starts to condense on the DMAE aggregates. Comparison to guiding quantum chemical calculations implies that now the het/hom spectral sequence is inverted and that the signals arise from a structure with mutual (m) OH⋯N or head-to-tail hydrogen bonding.^16,17^ It also implies that larger clusters must play a role for some of the unresolved broader band profile. In summary, there is spectral evidence for two hydrogen bond topologies of DMAE dimers, each with a homochiral and a heterochiral torsional variant. For the i topology, chirality synchronization appears to be less hindered by barriers than for the m topology. However, at least six different interconversion pathways are conceivable between the four conformations.

**Fig. 8:**
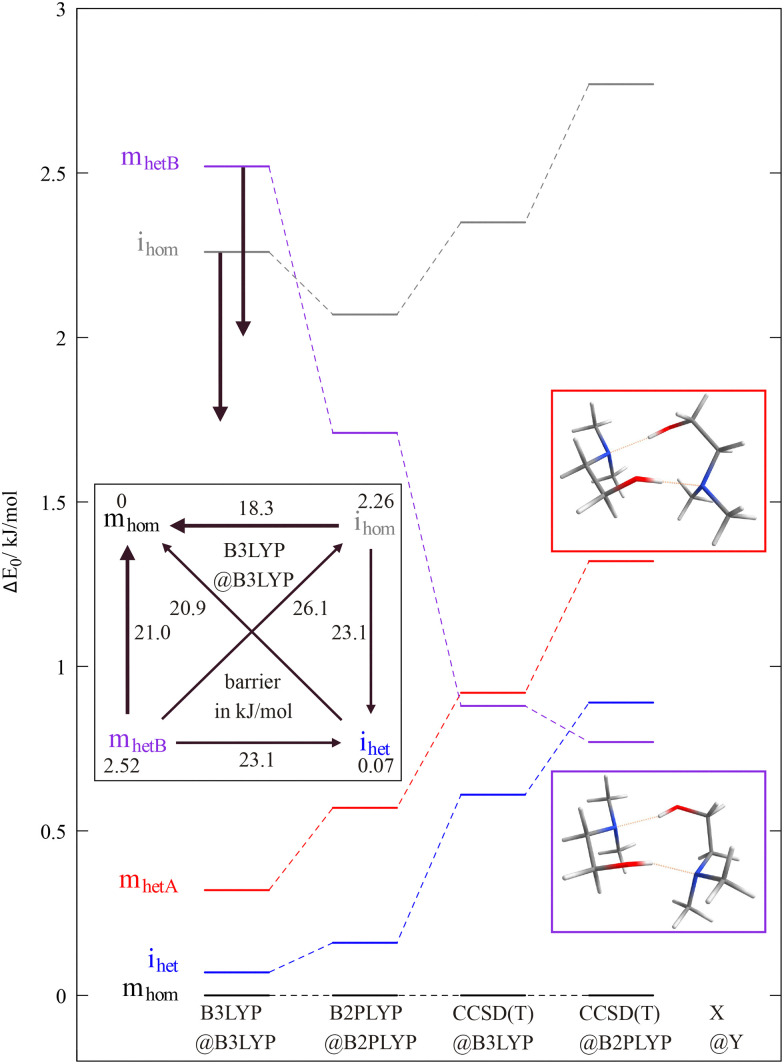
Relative energy of the hom(ochiral) and het(erochiral) dimers of DMAE as a function of electronic structure level X@Y (electronic energy at X level after structure optimisation at Y level, harmonic zero point energy added at Y level; X = CCSD(T) stands for DLPNO-CCSD(T)). Insertion (i) and mutual OHN hydrogen bonding (m) compete with each other. The relative energy of the two depicted m_het_ isomers depends strongly on the theory level and can serve as an experimental benchmark because they differ spectroscopically. The possible pathways for relaxation are depicted as an insert with zero-point-corrected barriers at B3LYP level. Thick arrows denote the most facile interconversions.

The conformational temperature of DMAE dimers generated in supersonic jet expansions can be estimated once electronic structure theory predicts reliable energies for the four detected isomers. This is far from straightforward, as Fig. 8 illustrates using the def2-QZVP basis set (for ma-def2-TZVP results, see ESI,† Fig. S21 and Tables S16, S17). For the homochiral (hom) pairing, all theory levels predict a relatively uniform energy disadvantage of 2–3 kJ mol^−1^ of the insertion (i) complex with a cooperative OH⋯OH⋯N pattern relative to the complex with isolated mutual OH⋯N hydrogen bonds (m). For the heterochiral (het) pairing, there are two variants of the m conformation (A and B). Variant A is consistently above the insertion complex by a small amount (≈0.5 kJ mol^−1^), whereas the variant B depends strongly on the level of calculation. At B3LYP level, m_hetB_ is 2.5 kJ mol^−1^ above insertion, whereas it becomes approximately isoenergetic at DLPNO-CCSD(T) level, thus winning over variant m_hetA_. The homochirality preference also increases with improving electronic structure level. B2PLYP is intermediate between B3LYP and DLPNO-CCSD(T) in most of its relative energy predictions. Therefore, any effective experimental conformational temperatures depend strongly on the level of theory used to estimate them. As interconversion between m_hetA_ and m_hetB_ is expected to be facile, only the lower one will be observed. Interconversion between i and m requires the breaking of hydrogen bonds and hom-het interconversion involves substantial torsional barriers. Therefore, it is plausible that up to four conformations survive in a supersonic jet expansion.

With this theoretical input, the assignments indicated in Fig. 7 were obtained, for details see the ESI,† Section S5.2 with Fig. S22 and Table S18. By computing barriers between the four conformations (see insert in Fig. 8 and Fig. S21 in the ESI†), a plausible relaxation picture for nitrogen as the carrier gas emerges. i_hom_ does not switch to i_het_ but instead interconverts preferentially to m_hom_, whereas m_het_ is more likely to switch relative monomer chirality without changing the hydrogen bond topology. i_het_ mainly survives because the driving force to relaxation towards m_hom_ is negligible. The associated barriers on the order of 20 kJ mol^−1^ can be overcome in the supersonic jet, because they either do not exist for separated monomers (i/m) or because the dimerisation process re-introduces sufficient internal energy into the cold monomers (hom/het).

### 5.4 The monohydrate of DMAE as a side benefit

**Fig. 9:**
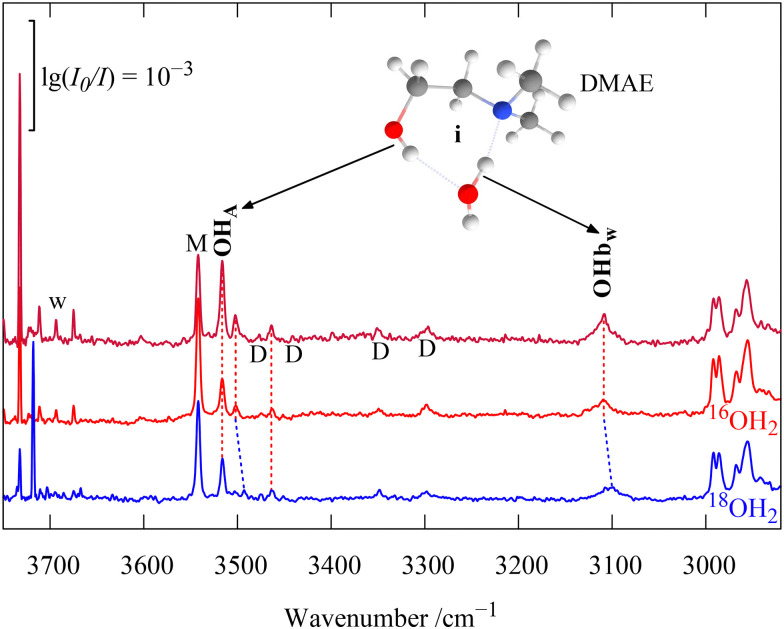
Detection of the inserted monohydrate of DMAE based on water concentration variation (red) and water ^18^O isotope substitution (blue spectrum). The more downshifted OH transition is due to the water vibration and the strongest of the three less shifted transitions is due to the alcoholic OH stretch. Connecting dashed lines show that the two weakest transitions behave differently with isotope substitution and might be due to an unassigned isomer. DMAE monomer (M) and dimer (D) signals are also marked. OHb_w_ is clearly a water vibration.

**Fig. 10:**
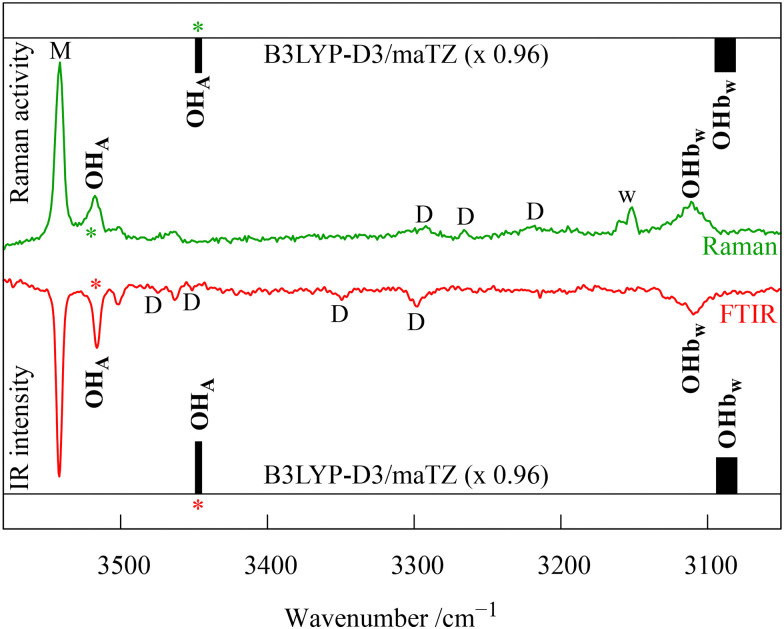
Comparison of Raman and (flipped) FTIR spectra of DMAE water co-expansions with 0.96× wavenumber-scaled harmonic predictions. Predicted relative intensities for the inserted 1 : 1 complex correspond to vertical bar areas (matched to the band intensities marked with a star). M, D and w mark DMAE monomer, dimer and water monomer transitions, respectively.

As pointed out in Fig. 7, traces of water in the DMAE expansion give rise to four small signals of DMAE hydrate complexes (marked +w). These are investigated more systematically in Fig. 9, extending to the water monomer and CH stretching regions. Addition of ^18^O-labelled water shows which bands are due to water OH stretching vibrations (tilted blue dashes). This is the case for the most downshifted and most pronounced signal (OHb_w_) and for a small feature at much higher wavenumber (close to 3500 cm^−1^). This points at a major and a minor isomer of DMAE monohydrate in the co-expansion, given their similar scaling with water concentration (Fig. 9). That is supported by the switch between carrier gases (Fig. 7), which affects all monohydrate signals similarly. We focus on the two intense transitions. The strongly downshifted and fairly broad water stretching signal must be due to an inserted complex, where it contacts the strong base and cooperatively acts as a hydrogen bond acceptor towards the alcoholic OH group, at the same time reducing strain. Its most intense counterpart at higher wavenumber is presumably due to the alcoholic OH stretch of DMAE. Harmonic DFT calculations suggest that the insertion complex is by far the most stable monohydrate and the results for DMAE dimers (Fig. 7) suggest that insertion is not kinetically hindered in the supersonic expansion. Note that a different behaviour has been assigned in a Ne matrix for the monohydrate of AE.^26^ The harmonic calculations for the insertion complex of the DMAE monohydrate predict the downshifted water vibration to be 2–3 times more intense than the alcoholic vibration, in both Raman and IR spectra. This fits the observation, as illustrated in Fig. 10, where theoretical intensities are illustrated with vertical bars. To account for the 3× larger width of the water vibration, the corresponding bar is drawn 3× as thick, while conserving its area.

From the spectra of DMAE monohydrate, a new HyDRA database entry for strongly hydrogen-bonded water complexes emerges.^42^ The OHb water vibration of interest at 3109(12) cm^−1^ involves no obvious anharmonic resonance (beyond the couplings which likely give rise to the full width at half maximum of about 15 cm^−1^) and thus has a likely spectroscopic purity *P* of >0.90. The situation at higher wavenumber is less clear, with a dominant alcohol vibration of the insertion complex and two minor contributions due to an isomer or perhaps (in the ^18^O-insensitive case) a resonance.

### 5.5 Performance and improvement of a HyDRA blind challenge model

One of the successful models in the first HyDRA challenge,^20^ a double-blind test for the predicting power of computational models for small to moderately downshifted OHb wavenumbers of a single water unit attached to organic molecules, was the NNB-HyDRA model (neural network-based Basel model for HyDRA).^43^ Here, this model is used for predicting the wavenumber of water inserted into the internal hydrogen bond of DMAE. The NNB-HyDRA model was trained in two stages. First, a map between structure and harmonic wavenumber was trained (base model), followed by a transfer learning (TL) step between harmonic wavenumbers and experimentally observed (training set) anharmonic wavenumbers. Transfer learning is a powerful technique to elevate base models to higher levels of theory or for model improvement using experimental data.^43,44^

Experimentally, the OH-stretch vibration for the DMAE monohydrate is considerably more downshifted than any of the molecules in the HyDRA data set (training and test). This more pronounced downshift arises due to the stronger intermolecular interactions. Since such strong intermolecular interactions are neither covered in the data set for the base model nor in the data set used for TL, it is not expected that NNB-HyDRA^43^ yields reliable results: the difference between measured and predicted anharmonic wavenumbers is Δ*ν* = 461 cm^−1^, see Table 1. This difference arises primarily as a consequence of the base model overestimating the harmonic wavenumber by Δ*ω* = 389 cm^−1^. To alleviate this and within the scope and logic of the approach,^43^ the structure of the DMAE monohydrate was optimised and the harmonic wavenumber was determined at the B3LYP-D3/aug-cc-pVTZ level of theory. Then, the base model was retrained (using 223 structures), followed by TL (using 9 structures) to yield NNB-HyDRA* which considerably improves the prediction (Δ*ν* = 177 cm^−1^).

Predictions of the NNB-HyDRA model for DMAE (*ν̃*_exp._ ∼ 3109 cm^−1^). NNB-HyDRA corresponds to the unaltered NNB-HyDRA model (base model was trained on roughly 222 molecules (9 train, 10 test, 203 others) and corresponding *ω*_OHb_, and transfer learned using 9 experimental wavenumbers (the radical, di-*tert*-butyl nitroxide, was omitted)) while NNB-HyDRA* corresponds to a model that also included the structure and harmonic wavenumber of DMAE to train the base model (note that the experimental DMAE wavenumber was not used for TL, *i.e.* the TL data set still consisted of 9 experimental wavenumbers). NNB-HyDRA*-VPT2 corresponds to a model for which the base model was trained on VPT2 instead of harmonic wavenumbers for a subset of the 223 molecules, followed by TL on the same 9 molecules. The proximity between *ν̃*^VPT2^_ref._ and *ν̃*_exp._ is probably coincidental. All wavenumbers are in cm^−1^

**Table:** 

Model	Base model			TL model	
	*ω*_pred._	*ω*_ref._	|Δ*ω*|	*ν̃*_pred._	|Δ*ν̃*|
NNB-HyDRA	3664.8	3275.8	389	3570.0	461
NNB-HyDRA*	3276.2	3275.8	0.4	3285.6	177

**Table:** 

Model	Base model			TL model	
	*ν̃* ^VPT2^_pred._	*ν̃* ^VPT2^_ref._	|Δ*ν̃*^VPT2^|	*ν̃*_pred._	|Δ*ν̃*|
NNB-HyDRA*-VPT2	3094.4	3093.4	1.0	3157.7	48.7

**Fig. 11:**
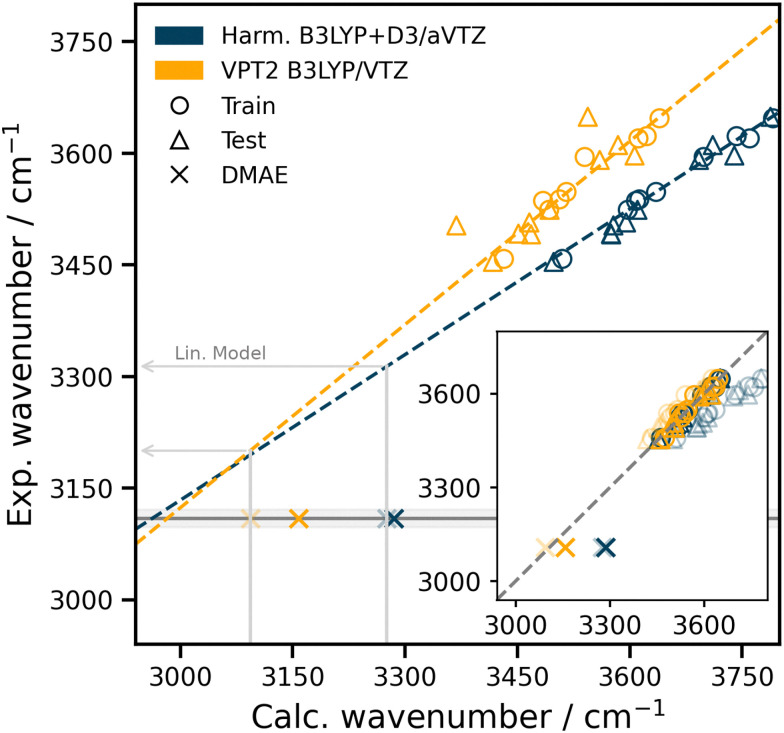
Main panel: Correlation between the DFT harmonic/VPT2 and experimental OH stretch wavenumbers for the training (circles) and test (triangle) sets of the HyDRA challenge. Dashed lines indicate linear regressions to the training set. Predictions for DMAE from NNB-HyDRA* and NNB-HyDRA*-VPT2 are shown as opaque ×, while the base model prediction (before TL) is shown as transparent ×. Inset: The correlation between the base model (transparent) and TL model predictions (opaque) for the HyDRA training and test sets. While the TL step clearly improves the predictions for the moderately downshifted OH-stretch wavenumbers (circles and triangles), they slightly worsen for DMAE (crosses). The dashed grey line is the 1 : 1 correlation.

The harmonic approximation, while computationally efficient, becomes inadequate when comparing with experimental observables in particular due to neglect of mechanical anharmonicity and/or coupling between modes. Consequently, geometry optimisations, harmonic frequency analyses, and VPT2 calculations were performed for 193 structures at a slightly reduced level of theory (B3LYP/cc-pVTZ), primarily to mitigate the substantial computational demands associated with the VPT2 treatment. Correlations between the measured and the DFT harmonic/VPT2 wavenumbers (blue/yellow) are shown in Fig. 11 together with linear regressions (blue and yellow dashed lines) to the training set (circles). The test sets (triangles) are shown for completeness. It is probably coincidental that these linear regressions cross close to where DMAE monohydrate is found experimentally, although one expects some degree of cancellation of anharmonic effects for strong hydrogen bonds, because diagonal anharmonicity increases and off-diagonal anharmonicity tends to compensate for this.^45^ The actual base predictions differ more (Table 1). It is found that for DMAE the VPT2 wavenumber (transparent × in the main panel) is much closer to the regression line (yellow). As the *ω*_ref._/*ν*^VPT2^_ref._ values define the quality of the base model from which the final NNB-model is obtained through TL, it is conceivable that a NNB-HyDRA*-VPT2 model more reliably extrapolates for strongly downshifted complexes. Indeed, evaluating the linear regression model at the calculated wavenumbers *ω*_ref._ = 3275.8 cm^−1^ and *ν*^VPT2^_ref._ = 3093.4 cm^−1^ yields estimates 3313.7 and 3200.3 cm^−1^, respectively, see grey lines in Fig. 11.

For an improved NNB-HyDRA*-VPT2 model the base model was retrained on the VPT2 data including DMAE, followed by TL on the original 9 training structures. For DMAE this new model predicts *ν*_pred._ = 3157.7 cm^−1^ with Δ*ν* = 49 cm^−1^ and considerably improves over the NNB-HyDRA* model for which Δ*ν* = 177 cm^−1^. With forthcoming reference data reporting more strongly downshifted 1 : 1 complexes a yet more robust NNB-HyDRA-VPT2 model can be trained in the future. Finally, model performance can be further improved by judicious choice of the size of the basis set and/or the level of theory for the reference data used to train the base model.

## 6 Conclusions

The compound class of internally hydrogen-bonded amino alcohols is seen to be suitable for the extraction of approximate effective Boltzmann temperatures for different degrees of freedom in mild supersonic jet expansions, bridging very cold vibrational jet spectra and room temperature band profiles in the OH stretching region. Rotational temperatures drop very quickly with stagnation pressure and show the expected dependence on the mass of the carrier gas, as long as cluster formation does not distort the picture. Boltzmann temperatures of low frequency vibrations which couple to the OH stretching motion by hydrogen bond strength modulation can be extracted in favourable cases, but there is significant uncertainty due to hot band contributions to the band profiles. Different degrees of conformational cooling can be observed for dimers of *N*,*N*-dimethylaminoethanol as a function of relative transient chirality. Because the computed energy differences – proportional to the conformational temperatures – exhibit significant uncertainties (Fig. 8), we refrain from extracting actual conformational temperatures from observed abundances.

To train more accurate quantum chemical approaches for such homodimers, we present benchmark OH stretching spectra of the monohydrate of *N*,*N*-dimethylaminoethanol, where the water inserts into the intramolecular hydrogen bond and is downshifted from its monomer symmetric stretching wavenumber by as much as 550 cm^−1^. This more than doubles the training range available for the previous OH stretching blind challenge HyDRA^20^ and can serve as a training data point for the next round of the challenge later this year, as illustrated in a case study for the neural network-based Basel model.^43^

Several observations on temperature-dependent spectra made in this work call for verification in related internally hydrogen-bonded hydrides. While some of them may be more suitable for rotational temperatures,^46^ others invite vibrational temperature analysis^47^ and still others may allow to follow conformational cooling^38^ as a function of soft expansion conditions. The new ability to combine such soft expansions with gas recycling technology helps to overcome the intrinsically low sensitivity of direct absorption and spontaneous Raman scattering approaches for the characterisation of the rarefied gas dynamics behind slit nozzles.

## Author contributions

E. L.: data curation, formal analysis, investigation (IR), visualisation, writing – review & editing; M. J. G.: data curation, formal analysis, investigation (IR), visualisation, writing – review & editing; N. O. B. L.: data curation, formal analysis, investigation (Raman), methodology, software, writing – review & editing; M. A. S.: conceptualisation, formal analysis, funding acquisition, methodology, supervision, writing – original draft; S. K.: conceptualisation, formal analysis, investigation (NNB), visualisation, writing – review & editing; V. A.: data curation, formal analysis, investigation (NNB), writing – review; M. B.: data curation, formal analysis, investigation (NNB), writing – review; M. M.: conceptualisation, formal analysis, funding acquisition, methodology, supervision, writing – original draft.

## Conflicts of interest

There are no conflicts to declare.

## Data availabilty

The spectroscopic data supporting this article have been included as part of the ESI.† In addition, the original Raman and infrared spectra and the .xyz files from the harmonic calculations are made available at the GRO.data repository at https://doi.org/10.25625/W5ZDOT. Raw DFT data and trained models for the DMAE monohydrate predictions are available at https://github.com/MMunibas/dmae.

## Supplementary Material

CP-027-D5CP02019K-s001

## References

[cit1] Snels M. , Horká-Zelenková V., Hollenstein H. and Quack M., in Handbook of High-resolution Spectroscopy, ed. M. Quack and F. Merkt, John Wiley & Sons, Ltd., Chichester, UK, 2011, vol. 2, pp. 1021–1067

[cit2] Puzzarini C., Stanton J. F., Gauss J. (2010). Int. Rev. Phys. Chem..

[cit3] Kjaergaard H. G., Robinson T. W., Howard D. L., Daniel J. S., Headrick J. E., Vaida V. (2003). J. Phys. Chem. A.

[cit4] Zischang J., Suhm M. A. (2014). J. Chem. Phys..

[cit5] Vogt E., Jensen C. V., Kjaergaard H. G. (2024). J. Phys. Chem. A.

[cit6] Maté B., Tejeda G., Montero S. (1998). J. Chem. Phys..

[cit7] Amirav A., Even U., Jortner J. (1980). Chem. Phys..

[cit8] Preuss D. R., Pace S. A., Gole J. L. (1979). J. Chem. Phys..

[cit9] Herrmann A., Hofmann M., Leutwyler S., Schumacher E., Wöste L. (1979). Chem. Phys. Lett..

[cit10] McClelland G., Saenger K., Valentini J., Herschbach D. (1979). J. Phys. Chem..

[cit11] Patterson D., Doyle J. M. (2012). Mol. Phys..

[cit12] Hutzler N. R., Lu H.-I., Doyle J. M. (2012). Chem. Rev..

[cit13] Thomsen D. L., Axson J. L., Schrøder S. D., Lane J. R., Vaida V., Kjaergaard H. G. (2013). J. Phys. Chem. A.

[cit14] Mulla S. T., Jose C. I. (1986). J. Chem. Soc., Faraday Trans. 1.

[cit15] Liu Y., Rice C. A., Suhm M. A. (2004). Can. J. Chem..

[cit16] Seurre N., Barbu-Debus K. L., Lahmani F., Zehnacker-Rentien A., Sepiol J. (2004). J. Mol. Struct..

[cit17] Asselin P., Madebène B., Soulard P., Georges R., Goubet M., Huet T. R., Pirali O., Zehnacker-Rentien A. (2016). J. Chem. Phys..

[cit18] Ager D. J., Prakash I., Schaad D. R. (1996). Chem. Rev..

[cit19] Lwin E., Lüttschwager N. O. B., Suhm M. A. (2025). Phys. Chem. Chem. Phys..

[cit20] Fischer T. L., Bödecker M., Schweer S. M., Dupont J., Lepère V., Zehnacker-Rentien A., Suhm M. A., Schröder B., Henkes T., Andrada D. M., Balabin R. M., Singh H. K., Bhattacharyya H. P., Sarma M., Käser S., Töpfer K., Vazquez-Salazar L. I., Boittier E. D., Meuwly M., Mandelli G., Lanzi C., Conte R., Ceotto M., Dietrich F., Cisternas V., Gnanasekaran R., Hippler M., Jarraya M., Hochlaf M., Viswanathan N., Nevolianis T., Rath G., Kopp W. A., Leonhard K., Mata R. A. (2023). Phys. Chem. Chem. Phys..

[cit21] Gottschalk H. C., Fischer T. L., Meyer V., Hildebrandt R., Schmitt U., Suhm M. A. (2021). Instruments.

[cit22] Lüttschwager N. O. B. (2024). Phys. Chem. Chem. Phys..

[cit23] Lüttschwager N. O. B. (2021). J. Open Source Software.

[cit24] Wang K., Miao T., Liu Y. (2011). Comput. Theor. Chem..

[cit25] Calabrese C., Maris A., Evangelisti L., Piras A., Parravicini V., Melandri S. (2018). Front. Chem..

[cit26] Yazdabadi S. H., Mihrin D., Feilberg K. L., Larsen R. W. (2024). J. Chem. Phys..

[cit27] Neese F. (2022). Wiley Interdiscip. Rev.: Comput. Mol. Sci..

[cit28] Herzberg G. , Molecular Spectra and Molecular Structure, D. Van Nostrand Company, vol. I, 1950

[cit29] Schreiber V., Melikova S., Rutkowski K., Shchepkin D., Shurukhina A., Koll A. (1996). J. Mol. Struct..

[cit30] Felder P., Günthard H. (1982). Chem. Phys..

[cit31] Poblotzki A., Gottschalk H. C., Suhm M. A. (2017). J. Phys. Chem. Lett..

[cit32] You A., Li Y., Yang X., Shen H., Yu Y., Zhou X., Zhang R., Liu S. (2025). J. Chem. Phys..

[cit33] Batista P. R., Karas L. J., Viesser R. V., de Oliveira C. C., Gonçalves M. B., Tormena C. F., Rittner R., Ducati L. C., de Oliveira P. R. (2019). J. Phys. Chem. A.

[cit34] Wassermann T. N., Suhm M. A. (2010). J. Phys. Chem. A.

[cit35] Fischer T. L., Jensen C. V., Lwin E., Pal D., Kjaergaard H. G., Suhm M. A. (2025). Phys. Chem. Chem. Phys..

[cit36] Howard D. L., Jørgensen P., Kjaergaard H. G. (2005). J. Am. Chem. Soc..

[cit37] Mackeprang K., Kjaergaard H. G., Salmi T., Hänninen V., Halonen L. (2014). J. Chem. Phys..

[cit38] Hartwig B., Suhm M. A. (2021). Phys. Chem. Chem. Phys..

[cit39] Lee J. J., Hesse S., Suhm M. A. (2010). J. Mol. Struct..

[cit40] Penn R., Buxton L. (1975). J. Mol. Spectrosc..

[cit41] Miller I., Thomas F., Clary D. C., Meijer A. J. H. M. (2005). J. Chem. Phys..

[cit42] Hydrate Donor Redshift Anticipation, a database maintained by the BENCh DFG research training group in Göttingen, 2024–2025, available under https://qmbench.net/databases/hydra

[cit43] Käser S., Töpfer K., Vazquez-Salazar L. I., Boittier E. D., Meuwly M. (2023). Phys. Chem. Chem. Phys..

[cit44] Käser S., Boittier E. D., Upadhyay M., Meuwly M. (2021). J. Chem. Theory Comput..

[cit45] Heger M., Andersen J., Suhm M. A., Wugt Larsen R. (2016). Phys. Chem. Chem. Phys..

[cit46] Sharma A., Reva I., Fausto R., Hesse S., Xue Z., Suhm M. A., Nayak S. K., Sathishkumar R., Pal R., Guru Row T. N. (2011). J. Am. Chem. Soc..

[cit47] Hesse S., Wassermann T. N., Suhm M. A. (2010). J. Phys. Chem. A.

