# Supplementary material for: Rotational, vibrational, conformational and diastereomeric dimer cooling of aminoalcohols in soft supersonic expansions and the monohydrate of dimethylaminoethanol
Source: Phys Chem Chem Phys. 2025 Jul 24;27(34):17692–703. doi: 10.1039/d5cp02019k (PMC12308818; doi:10.1039/d5cp02019k)
Supplement: CP-027-D5CP02019K-s001 [file CP-027-D5CP02019K-s001.pdf]

Supplementary Information (SI) for Physical Chemistry Chemical Physics.
This journal is © the Owner Societies 2025

Rotational, vibrational, conformational and diastereomeric dimer cooling of aminoalcohols in soft supersonic expansions and the monohydrate of dimethylaminoethanol

Eaindra Lwin, Mathis J. Gölz, Nils O. B. Lüttschwager, Martin A. Suhm*

Institute of Physical Chemistry, University of Göttingen, Tammannstr. 6, 37077 Göttingen (Germany). E-mail: msuhm@gwdg.de

Silvan Käser, Valerii Andreichev, Magalie A. Brandes, and Markus Meuwly*

Department of Chemistry, University of Basel, Klingelbergstr. 80, CH-4056 Basel, Switzerland. E-mail: m.meuwly@unibas.ch

Contents

1 Experiment **S3**
- 1.1 Investigated compounds . . . S3
- 1.2 Experimental details . . . S3

2 Rotational temperatures **S7**
- 2.1 Spectral series for T_r . . . S10
- 2.2 Fitting parameters for T_r . . . S14

3 Vibrational temperatures **S21**
- 3.1 Integration method . . . S23
- 3.2 Vibrational temperature of ON and Wag . . . S25

4 Modelling **S26**
- 4.1 Details on harmonic calculations . . . S26
- 4.2 Example inputs for NEB scans . . . S26

5 Spectral assignments **S28**
- 5.1 Nomenclature . . . S28
- 5.2 Dimethylaminoethanol (DMAE) . . . S28
- 5.3 Aminoethanol (AE) . . . S33
- 5.4 Methylaminoethanol (MAE) . . . S35
- 5.5 Dimethylaminopropanol (DMAP) . . . S38
- 5.6 Comparison of investigated compounds . . . S38

6 Entries for the HyDRA database **S40**

References **S41**

List of Tables

- S1 Table of investigated compounds . . . S3
- S2 Experimental details for the FTIR spectra of DMAE and AE . . . S4
- S3 Experimental details for the FTIR spectra of DMAP, MAE, and DMAE with water . . . S5
- S4 Experimental details for Raman spectra . . . S6
- S5 Overview of the results for the rotational temperature calculations for DMAE . . . S8
- S6 Overview of the results for the rotational temperature calculations for AE . . . S9
- S7 Experimental band centers $\tilde{\nu}$ and IR intensities I of DMAE . . . S23
- S8 Experimental band center $\tilde{\nu}$ and IR intensities I of DMAP . . . S24
- S9 Corrected intensities I of DMAP . . . S24

S10 Vibrational temperature T_v of ON in DMAE . . . S25
S11 Vibrational temperature T_v of ON in DMAP . . . S25
S12 Vibrational temperature T_v of Wag in DMAP . . . S25
S13 Example input for NEB scan . . . S26
S14 Example input for transition state calculation . . . S27
S15 Nomenclature for spectral features . . . S29
S16 Relative energy for different DMAE dimers using the def2-QZVP basis set . . . S30
S17 Relative energy for different DMAE dimers using the ma-def2-TZVP basis set . . . S30
S18 DMAE computational results . . . S32
S19 AE computational results OH vibrations . . . S34
S20 AE computational results NH vibrations . . . S34
S21 Harmonic MAE monomer predictions . . . S35
S22 Relative energy for different MAE conformers . . . S36
S23 DMAP computational results . . . S38
S24 Comparison of experimental and scaled theory predictions . . . S40
S25 HyDRA database entries . . . S40

List of Figures

S1 Schematic picture of the *gratin* jet spectrometer . . . S3
S2 Spectral series for T_r - DMAE 1 hPa in He . . . S10
S3 Spectral series for T_r - DMAE 1 hPa in Ne . . . S11
S4 Spectral series for T_r - DMAE 1 hPa in N_2 . . . S11
S5 Spectral series for T_r - DMAE 5 hPa in He . . . S12
S6 Spectral series for T_r - DMAE 5 hPa in Ne . . . S12
S7 Spectral series for T_r - AE 0.4 hPa in He . . . S13
S8 Spectral series for T_r - AE 0.4 hPa in Ne . . . S13
S9 Rotational temperature fitting for 1 hPa DMAE in He . . . S14
S10 Rotational temperature fitting for 1 hPa DMAE in Ne . . . S15
S11 Rotational temperature fitting for 1 hPa DMAE in N_2 . . . S16
S12 Rotational temperature fitting for 5 hPa DMAE in He . . . S17
S13 Rotational temperature fitting for 5 hPa DMAE in Ne . . . S18
S14 Rotational temperature fitting for 0.4 hPa AE in He . . . S19
S15 Rotational temperature fitting for 0.4 hPa AE in Ne . . . S20
S16 Four state model for vibrational temperatures . . . S21
S17 Sum and difference bands in FTIR spectra . . . S22
S18 Intermolecular interactions in amino alcohol dimers . . . S28
S19 Dihedral NCCO angle of aminoethanols . . . S28
S20 Structures of the DMAE dimers . . . S29
S21 Relative energy of the hom and het dimers of DMAE using the ma-def2-TZVP basis set . . . S31
S22 Raman and FTIR spectra of DMAE . . . S32
S23 Raman and FTIR spectra of AE . . . S33
S24 Raman and FTIR spectra of MAE . . . S35
S25 Energy barrier between trans (t) and gauche (g) MAE . . . S36
S26 IR spectra of MAE for different expansion conditions . . . S37
S27 Raman and FTIR spectra of DMAP . . . S38
S28 Comparison of FTIR spectra . . . S39

1 Experiment

1.1 Investigated compounds

Tab. S1 gives detailed information about the chemicals used for the study and introduces the codes for the chemical names that are also used in the main document.

Table S1: Table of investigated chemicals, the introduced code names (supplement and main document), their CAS number, the supplier, purity, Lot# and the approximate (estimated) vapor pressure at 20°C.

Name	Code	CAS Number	Supplier	Purity	Lot#	vapour pressure/ hPa
aminoethanol	AE	141-43-5	TCI	>99.0%	LD8MN-OZ	0.5[1]
methylaminoethanol	MAE	109-83-1	TCI	>99.0%	A009803101	0.7[2]
dimethylaminoethanol	DMAE	108-01-0	TCI	99.996%	SJU3C-XX	6.1[3]
dimethylaminopropanol	DMAP	3179-63-3	TCI	98.0%	WQXEC-XR	1.6[4]
Demineralised water	H_2O	7732-18-5	-	-	-	
Helium	He	7440-59-7	Nippon	99.996%	-	
Neon	Ne	7440-59-7	Linde	99.999%	-	
Nitrogen	N2	7727-37-9	Nippon	99.999%	-	

1.2 Experimental details

All FTIR spectra were obtained using a *g*as *r*ecycling *at*om economic *in*frared (*gratin*) jet spectrometer (for a detailed description see [5]). To perform the soft supersonic jet expansions, the *gratin* jet spectrometer is modified by introducing a Pfeiffer Adixen ACP 40CP pump, shown in Fig. S1, to replace the previous function of a Busch Cobra NS 0070 C screw pump, which cannot handle exhaust pressures below 300 hPa. The ACP 40CP pump can handle any inlet pressure that is compatible with the Okta pumps and any outlet pressure up to 1 atmosphere needed. This allows for soft supersonic expansions with low stagnation pressures, thus enabling the exploration of a wider range of expansion temperatures.
All important parameters for the recorded FTIR spectra can be found in Table S2 and S3. The spectral traces are provided here: dataset.

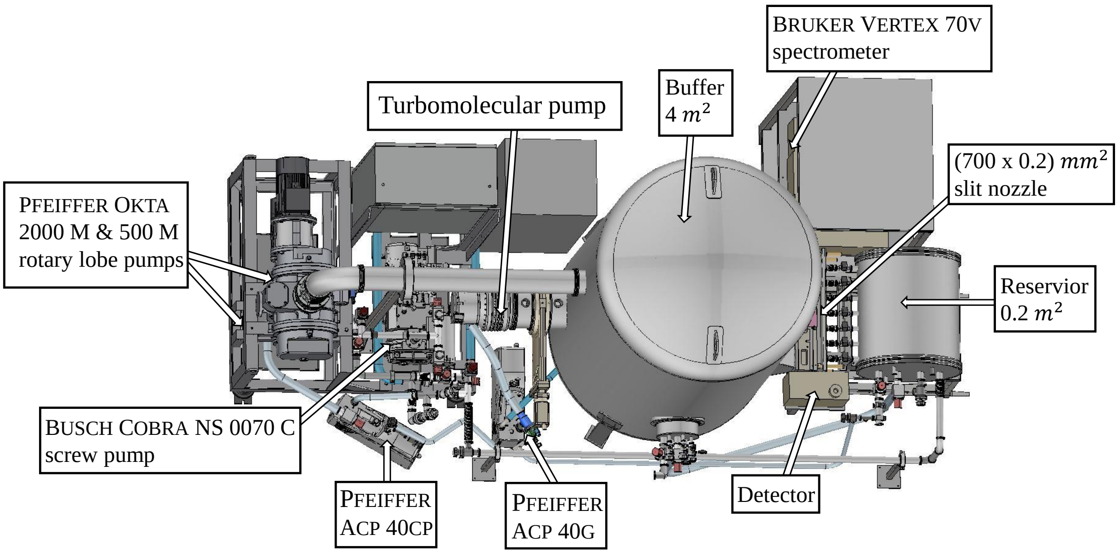

Figure S1: Schematic picture of the *gratin* jet spectrometer, created by Reinhard Hildebrandt from the institute mechanics workshop. The Pfeiffer ACP 40CP multistage roots pump was replaced by a Leybold Ecodry Plus 65 multistage roots pump with comparable features in August 2024. All experiments after 14.08.2024 are done by using the Leybold Ecodry Plus 65 in the recycling circuit.

Table S2: Spectroscopic details for the figures 2, 6 and 7 in the main document and S2, S3, S4, S5, S6, S7, S8, S22 and S23 from the supplementary information. The partial pressures of the amino alcohol (p_A), as well as the carrier gases helium (p_{He}), nitrogen (p_{N_2}) and neon (p_{Ne}) and the total stagnation pressure of the expansion (p_s) are given. The spectra were obtained by averaging # FTIR scans during 133 ms gas pulses through a 700 mm×0.2 mm slit nozzle with a Bruker VERTEX 70v FTIR spectrometer in double-sided mode at 140 kHz scanning speed. A 20 W tungsten light source, an InSb/HgCdTe sandwich detector and an optical filter (wavenumber range <4000 cm^{-1}) were used. The date of the spectrum being recorded is given in dd/mm/yyyy format and in the last column it is indicated in which figure(s) in the main publication and ESI the corresponding spectrum is shown. All the spectra are provided here: dataset.

p_A / hPa	p_{He} / hPa	p_{N_2} / hPa	p_{Ne} / hPa	p_s / hPa	#	dd/mm/yyyy	figure(s)
DMAE							
1	0	0	0	0	—	11/04/2024	2, 6, S2, S3, S4, S5, S6
5	0	0	15	20	100	09/04/2024	2, 6, S6
5	0	0	45	50	200	09/04/2024	2, S6
5	0	0	95	100	100	09/04/2024	2, S6
5	0	0	195	200	100	09/04/2024	2, S6
5	0	0	395	400	100	09/04/2024	2, S6
1	0	0	19	20	200	10/04/2024	S3
1	0	0	49	50	200	10/04/2024	S3
1	0	0	99	100	100	10/04/2024	S3
1	0	0	199	200	100	10/04/2024	S3
1	0	0	399	400	100	10/04/2024	S3
5	5	0	0	10	100	11/04/2024	S5
5	15	0	0	20	100	11/04/2024	6, S5
5	25	0	0	30	200	18/04/2024	S5
5	45	0	0	50	200	18/04/2024	S5
5	95	0	0	100	100	18/04/2024	S5
5	195	0	0	200	100	18/04/2024	S5
5	395	0	0	400	100	18/04/2024	S5
1	0	0	0	1	300	23/05/2024	S2, S3, S4
5	0	0	0	5	300	23/05/2024	S5,S6
1	9	0	0	10	100	30/05/2024	7, S2
1	19	0	0	20	100	30/05/2024	6, S2
1	49	0	0	50	100	30/05/2024	7, S2
1	99	0	0	100	100	30/05/2024	7, S2
1	199	0	0	200	100	30/05/2024	S2
1	299	0	0	300	50	30/05/2024	S2
1	399	0	0	400	50	30/05/2024	7, S2
1	0	9	0	10	100	16/12/2024	S4
1	0	19	0	20	100	16/12/2024	S4
1	0	49	0	50	200	16/12/2024	7, S4
1	0	99	0	100	100	16/12/2024	S4
1	0	199	0	200	100	16/12/2024	7, S4
1	0	299	0	300	100	16/12/2024	S4
1	0	399	0	400	100	16/12/2024	S4
0.2	0	0	400	400	200	17/04/2024	7, S22
AE							
0.4	0	0	0	0	—	25/04/2024	S7, S8
0.4	0	0	20	20.4	200	24/04/2024	S8
0.4	0	0	50	50	100	24/04/2024	S8
0.4	0	0	100	100	100	24/04/2024	S8
0.4	0	0	400	400	100	24/04/2024	S8
0.4	20	0	0	20.4	200	25/04/2024	S7
0.4	50	0	0	50	100	25/04/2024	S7
0.4	70	0	0	70	100	25/04/2024	S7
0.4	100	0	0	100	100	25/04/2024	S7
0.4	400	0	0	400	100	25/04/2024	S23
0.2	0	0	400	400	400	29/04/2024	S23

Table S3: Spectroscopic details for the figures 6, 9 and 10 in the main document and S17, S24, S26 and S27 from the supplementary information. The partial pressures of the amino alcohol (p_A), any added solvent (p_A), as well as the carrier gases helium (p_{He}), nitrogen (p_{N_2}) and neon (p_{Ne}) and the total stagnation pressure of the expansion (p_s) are given. The spectra were obtained by averaging # FTIR scans during 133 ms gas pulses through a 700 mm×0.2 mm slit nozzle with a Bruker VERTEX 70v FTIR spectrometer in double-sided mode at 140 kHz scanning speed. A 20 W tungsten light source, an InSb/HgCdTe sandwich detector and an optical filter (wavenumber range <4000 cm^{-1}) were used. The date of the spectrum being recorded is given in dd/mm/yyyy format and in the last column it is indicated in which figure(s) in the main publication the corresponding spectrum is shown. The spectral traces are provided here: dataset.

p_A / hPa	p_{He} / hPa	p_{N_2} / hPa	p_{Ne} / hPa	p_s / hPa	#	dd/mm/yyyy	figure(s)
DMAP							
1	0	0	0	0	—	06/06/2024	6, S17
1.5	8.5	0	0	10	100	10/06/2024	6
1.5	18.5	0	0	20	100	10/06/2024	6
1	49	0	0	50	200	06/06/2024	S17
0.2	0	0	400	400	400	03/06/2024	S17, S27
MAE							
0.85	0	0	10	11	150	21/05/2024	S26
0.85	0	0	30	31	100	21/05/2024	S26
0.1	0	0	400	400	400	17/05/2024	S26
0.4	0	0	400	400	200	16/05/2024	S24
0.2	0	400	0	400	200	14/02/2025	S26
0.2	0	600	0	600	100	14/02/2025	S26

p_A / hPa	p_S / hPa	p_{He} / hPa	p_{Ne} / hPa	p_s / hPa	#	dd/mm/yyyy	figure(s)
DMAE	H_2O						
0.05	0.1	0	400	400	1400	02/07/2024 + 22/10/2024	9, 10
0.05	0.3	0	400	400	800	13/04/2025	9
DMAE	$^{18}OH_2$						
0.05	0.1	0	400	400	1500	12/09/2024 + 21/10/2024	9

The Raman spectra that are shown in this work were measured using the *cookie-jet* setup, a *co*mpact, *c*o*st-efficient Raman wor*k*stat*i*on for mol*e*cular jet*-spectroscopy. This recycling setup is described in detail in reference [6]. All spectra were measured from continuous expansions through a 4 mm × 0.15 mm slit nozzle using a 532 nm solid-state laser with 25 W maximum optical output power (used at 20 W) for Raman excitation. Evacuation of the measurement cell and compression towards the nozzle was done by a dry-compressing, multi-stage roots pump with 55 m^{3}h^{-1} pumping speed (Leybold, Ecodry 65, *circulation pump*). Sample preparation was done by mixing the substance(s) and carrier gas into the measurement cell with the circulation pump switched off (pressures p_A, p_{He}, and p_{Ne} in Table S4). Subsequent circulation yielded pressure gradients of a factor of about 50 to 100 (p_s/p_b, see Table S4). Note that due to adsorption of the substances on surfaces in the measurement cell and vacuum setup (and suspected thermal decomposition in the hot circulation pump in case of AE), the concentrations calculated from partial pressures of substance and carrier gas are only approximate. In case of the DMAE measurement with addition of water (first row in Table S4), the water amount would drop during the measurements, which was counteracted by letting further water vapour into the measurement cell while the circulation was running. Therefore, no amount of water is specified. However, judging from the intensity of the water monomer band and CH stretching band of DMAE, we estimate that water was present in considerable excess to DMAE. Also for the DMAE plus water measurement, note that the setup was equipped with an additional 6 L buffer volume on the low pressure side of the circulation pump, leading to a stronger compression from 50 mbar (p_{He}, static) to 500 mbar (p_s, dynamic) when circulating the gas sample.

Table S4: Experimental details for Raman spectra. The partial pressures of the amino alcohol (p_A), as well as the carrier gases helium (p_{He}) and neon (p_{Ne}) and the total stagnation pressure of the expansion (p_s) as well as the background pressure in the measurement cell p_b are given. d denotes the distance between the laser beam and the nozzle, t the overall exposure time. The spectral traces are provided here: dataset.

p_A / hPa	p_{He} / hPa	p_{Ne} / hPa	p_s / hPa	p_b / hPa	d_{nozzle} / mm	t / hr:min	dd/mm/yyyy	Figure
DMAE								
0.05†	50	—	500	9	1	2:00	2025-04-17	10
0.2	100	—	500	9	2	10:00	2024-06-03	S22
AE								
0.2	—	82	480	6	1	1:00	2024-06-18	S23
MAE								
0.2	—	88	510	6	1	1:50	2024-07-02	S24
DMAP								
0.2	—	80	480	6	1	2:00	2024-07-09	S27

†water vapour added

2 Rotational temperatures

To estimate rotational temperatures T_r, equation S1 was used. It is based on a formula for the band profile of diatomic molecules[7], but the quadratic dependence should be approximately valid for the band contour of other rotors.

$$T_\mathrm{r} = \left(\frac{\Delta\nu_\mathrm{PQ}}{C}\right)^2 \tag{S1}$$

Here, C is a calibration constant determined at laboratory temperature, where the rotational temperature is in full equilibrium with other degrees of freedom, and $\Delta\nu_\mathrm{PQ}$ is the half width at half maximum (HWHM) of the fundamental OH-stretching vibration band at given conditions. Calibration constants amount to 0.5331 and 1.027 for DMAE and AE, respectively.

The given description is used to obtain upper limits for the rotational temperatures $T_\mathrm{r,high}$, where we assume that the fundamental band is infinitely narrow at 0 K. To estimate a lower limit for rotational temperatures, we subtract the lowest HWHM out of all measurements up to 400 hPa, corresponding to the rotationally coldest measurement, from all other measured HWHM. Additivity of HWHM values would be given for Lorentzian line profiles, but can be approximately assumed in the present estimates as well. In this approximation, the coldest achieved rotational temperature would be 0 K. DMAE and AE were studied separately. The revised lower limit calibration constants amount to 0.4013 and 0.8174 for DMAE and AE, respectively.

The figures 4 and 5 in the main document show the average rotational temperature (between the upper and lower limit), which will be called T_r in the following. The error bars shown span the upper limit and lower limit values for T_r. Details about the estimation of rotational temperatures can be found in Table S5 and S6.

Table S5: Overview of the results for the rotational temperature calculations for DMAE. The table lists the partial pressure of DMAE p_{DMAE}, the carrier gas, the stagnation pressure p_{s}, the half width at half maximum HWHM, the lower and higher limit rotational temperature $T_{\mathrm{r,low}}$ and $T_{\mathrm{r,high}}$, and the average rotational temperature T_{r}. For the gas phase spectrum, the stagnation pressure is approximately zero. For this analysis, the highest intensity peak of the coldest measurement (3541.82 cm^{-1}) was used as the Q branch, and the HWHM was determined at the side of the P branch.

p_{DMAE} / hPa	carrier gas	p_{s} / hPa	HWHM / cm^{-1}	$T_{\mathrm{r,low}}$ / K	$T_{\mathrm{r,high}}$ / K	T_{r} / K
5	helium	0	9.2	295	295	295
5	-	5	7.9	200	222	211
5	helium	10	7.8	189	213	201
5	helium	20	7.1	144	176	160
5	helium	30	6.6	116	153	134
5	helium	50	5.9	84	124	104
5	helium	100	5.0	48	90	69
5	helium	200	4.2	22	61	42
5	helium	400	3.4	8	41	24
5	neon	20	6.3	103	141	122
5	neon	50	5.3	58	99	79
5	neon	100	4.9	43	84	63
5	neon	200	4.1	21	59	40
5	neon	400	3.3	6	37	22
1	-	1	8.9	271	277	274
1	helium	10	7.6	180	206	193
1	helium	20	6.8	130	164	147
1	helium	50	4.9	44	86	65
1	helium	100	3.7	12	47	30
1	helium	200	2.8	2	28	15
1	helium	300	2.6	1	23	12
1	helium	400	2.5	0	21	11
1	nitrogen	10	6.5	114	150	132
1	nitrogen	20	5.4	62	104	83
1	nitrogen	50	4.6	33	73	53
1	nitrogen	100	4.3	25	64	45
1	nitrogen	200	4.0	19	57	38
1	nitrogen	300	4.2	23	62	42
1	nitrogen	400	4.2	23	61	42
1	neon	20	5.4	62	103	82
1	neon	50	3.9	16	52	34
1	neon	100	3.2	6	36	21
1	neon	200	2.7	1	25	13
1	neon	400	2.5	0	21	11
0.2	neon	400	2.3	0	18	9

Table S6: Overview of the results for the rotational temperature calculations for AE. The table lists the partial pressure of AE p_{AE}, the carrier gas, the stagnation pressure p_{s}, the half width at half maximum HWHM, the lower and higher limit rotational temperature $T_{\mathrm{r,low}}$ and $T_{\mathrm{r,high}}$, and the average rotational temperature T_{r}. For the gas phase spectrum, the stagnation pressure is approximately zero. For this analysis, the highest intensity peak of the coldest measurement (3568.81 cm^{-1}) was used as the Q branch, and the HWHM was determined at the side of the P branch.

p_{AE} / hPa	carrier gas	p_{s} / hPa	HWHM / cm^{-1}	$T_{\mathrm{r,low}}$ / K	$T_{\mathrm{r,high}}$ / K	T_{r} / K
0.4	helium	0	17.6	295	295	295
0.4	helium	10	12.3	113	143	128
0.4	helium	20	10.4	69	103	86
0.4	helium	50	7.3	20	50	35
0.4	helium	70	6.1	9	35	22
0.4	helium	100	5.4	5	28	16
0.4	helium	400	3.8	0	14	7
0.4	neon	5	15.2	203	220	211
0.4	neon	10	10.2	66	99	82
0.4	neon	20	7.7	25	57	41
0.4	neon	50	5.8	7	32	19
0.4	neon	100	4.4	1	19	10
0.4	neon	400	3.6	0	12	6

2.1 Spectral series for T_r

Spectral series similar to the one displayed in Fig. 1 of the main document are shown, all scaled to the same peak height.

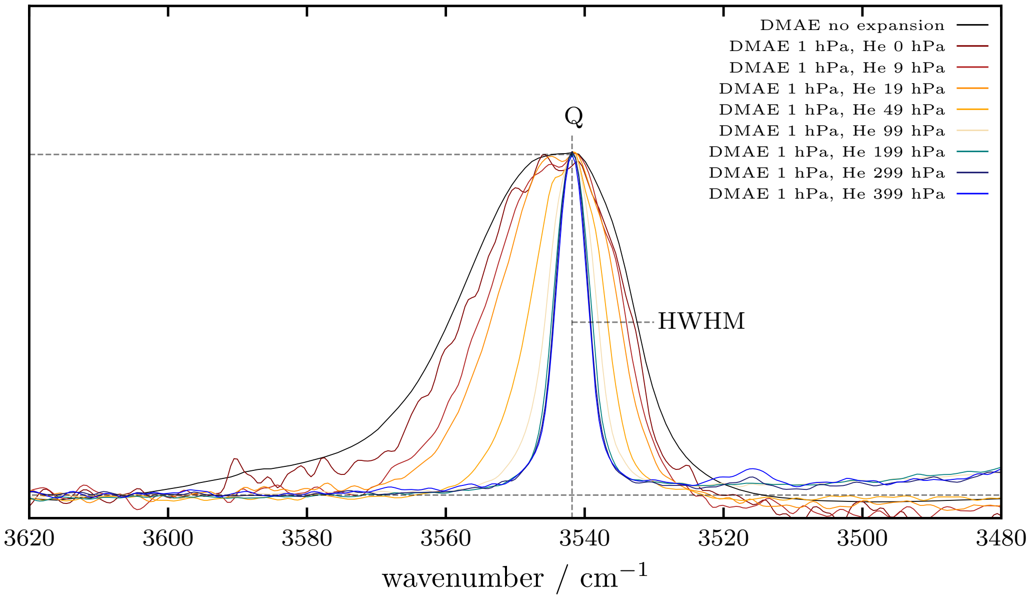

Figure S2: Evolution of the OH stretching fundamental for 1 hPa DMAE with the addition of increasing amounts of carrier gas (helium) to the expansion, scaled to the same peak height. Also shown is the stagnant gas phase spectrum, which serves as a temperature reference. The low-frequency half-width at half-maximum is used for a rotational temperature estimate.

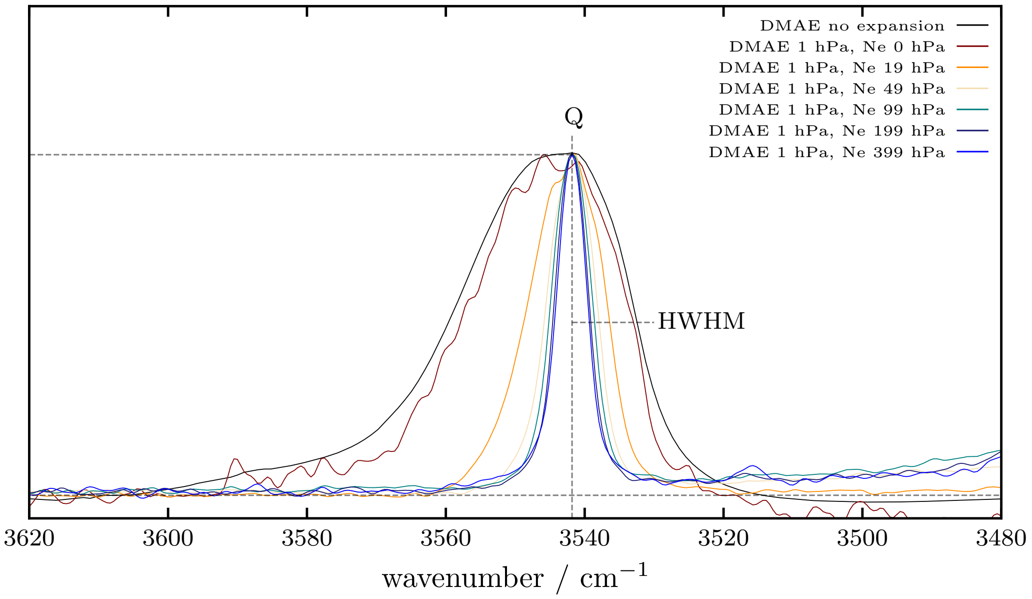

Figure S3: Evolution of the OH stretching fundamental for 1 hPa DMAE with the addition of increasing amounts of carrier gas (neon) to the expansion, scaled to the same peak height. Also shown is the stagnant gas phase spectrum, which serves as a temperature reference. The low-frequency half-width at half-maximum is used for a rotational temperature estimate.

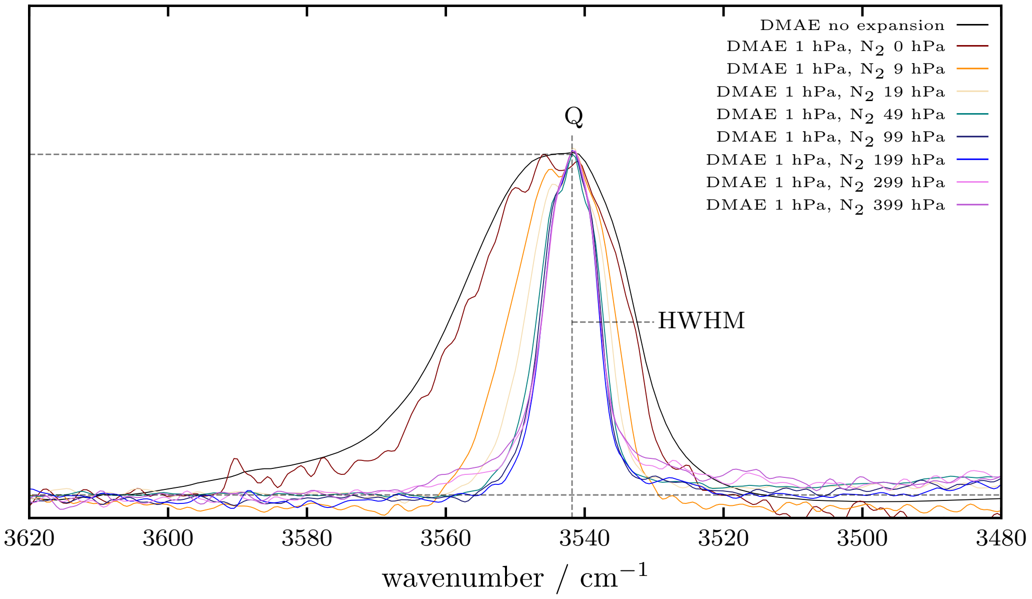

Figure S4: Evolution of the OH stretching fundamental for 1 hPa DMAE with the addition of increasing amounts of carrier gas (nitrogen) to the expansion, scaled to the same peak height. Also shown is the stagnant gas phase spectrum, which serves as a temperature reference. The low-frequency half-width at half-maximum is used for a rotational temperature estimate.

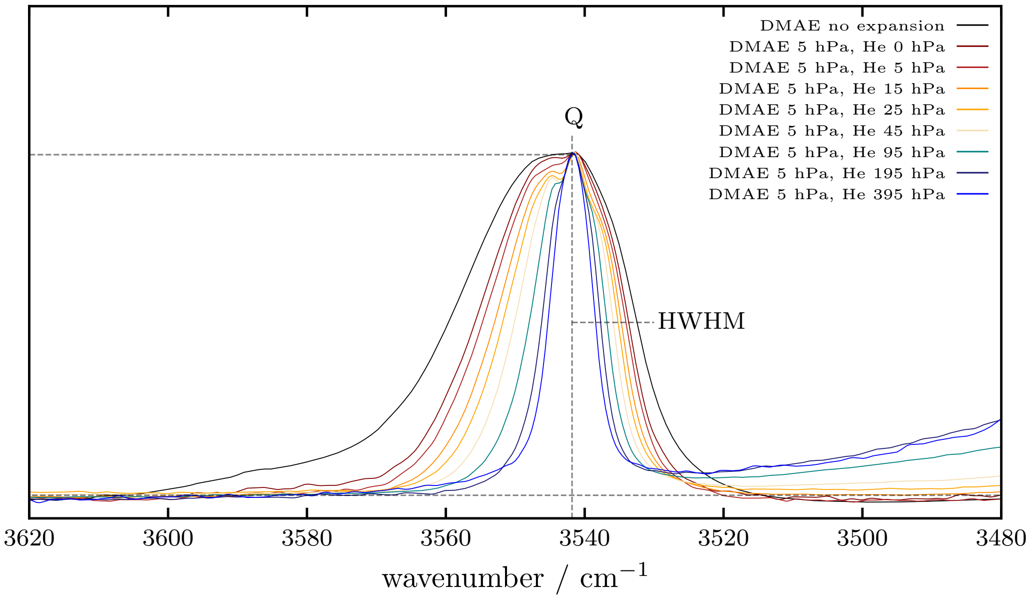

Figure S5: Evolution of the OH stretching fundamental for 5 hPa DMAE with the addition of increasing amounts of carrier gas (helium) to the expansion, scaled to the same peak height. Also shown is the stagnant gas phase spectrum, which serves as a temperature reference. The low-frequency half-width at half-maximum is used for a rotational temperature estimate.

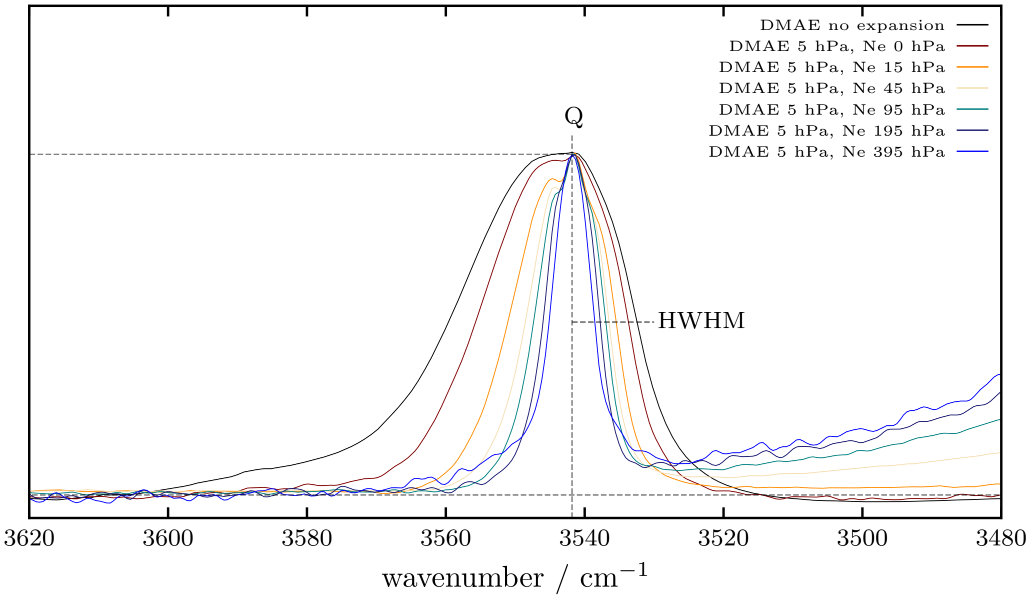

Figure S6: Evolution of the OH stretching fundamental for 5 hPa DMAE with the addition of increasing amounts of carrier gas (neon) to the expansion, scaled to the same peak height. Also shown is the stagnant gas phase spectrum, which serves as a temperature reference. The low-frequency half-width at half-maximum is used for a rotational temperature estimate.

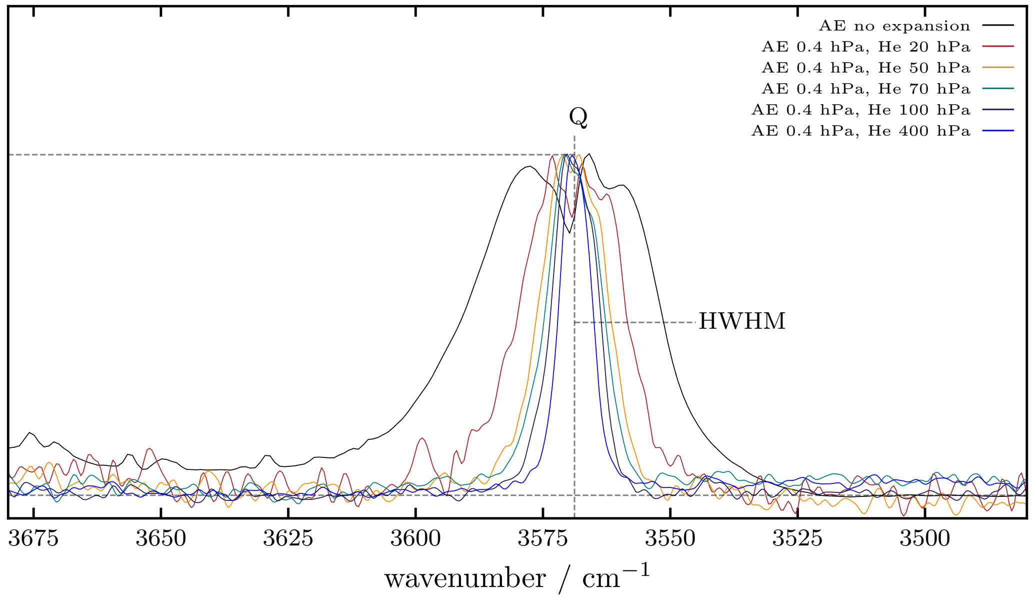

Figure S7: Evolution of the OH stretching fundamental for 0.4 hPa AE with the addition of increasing amounts of carrier gas (helium) to the expansion, scaled to the same peak height. Also shown is the stagnant gas phase spectrum, which serves as a temperature reference. The low-frequency half-width at half-maximum is used for a rotational temperature estimate.

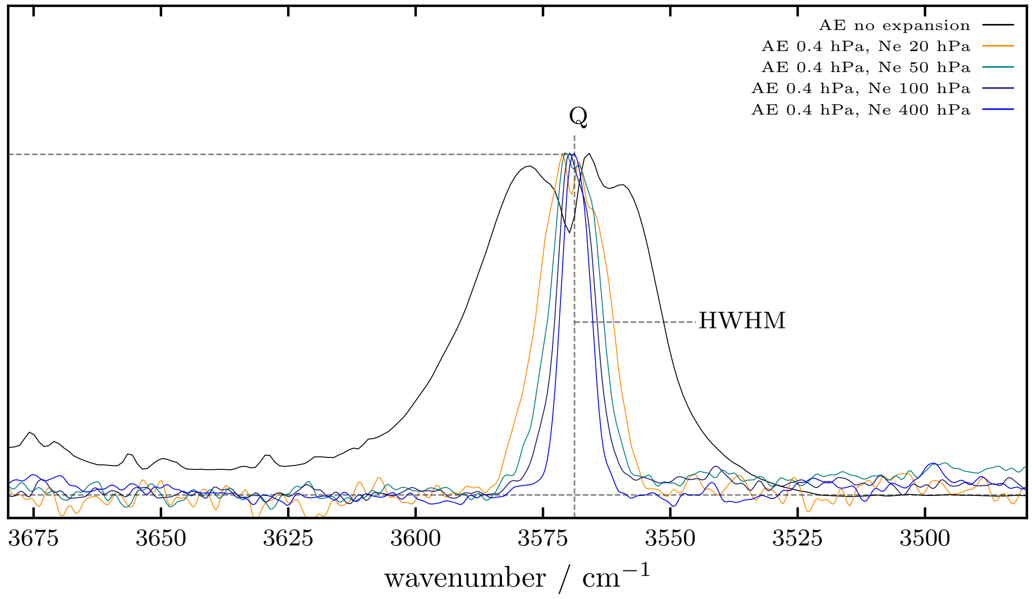

Figure S8: Evolution of the OH stretching fundamental for 0.4 hPa AE with the addition of increasing amounts of carrier gas (neon) to the expansion, scaled to the same peak height. Also shown is the stagnant gas phase spectrum, which serves as a temperature reference. The low-frequency half-width at half-maximum is used for a rotational temperature estimate.

2.2 Fitting parameters for T_r

For the fitting of the rotational temperature T_r, we assumed the temperature extracted at 400 hPa stagnation pressure as the coldest one (T_∞). Without expansion, the stagnation temperature corresponds to the laboratory temperature ($T_s = 295$K). The applied fitting function $\Phi(T_r) = \ln(T_r/T_\infty)/\ln(T_s/T_\infty)$ is 1 when $T_r = T_s$, and 0 when $T_r = T_\infty$. The steeper the curve is, the more efficient the rotational cooling is. The rotational temperature T_r is found to exponentially decrease with the stagnation pressure with the function $\Phi(T_r) = \exp(-(p_s/\mathrm{hPa})^{3/4}/c)$, where c is a measure of the cooling efficiency and the exponent 3/4 is obtained by trial and error. The smaller the fitted c parameter, the steeper the curve and the more efficient rotational cooling.

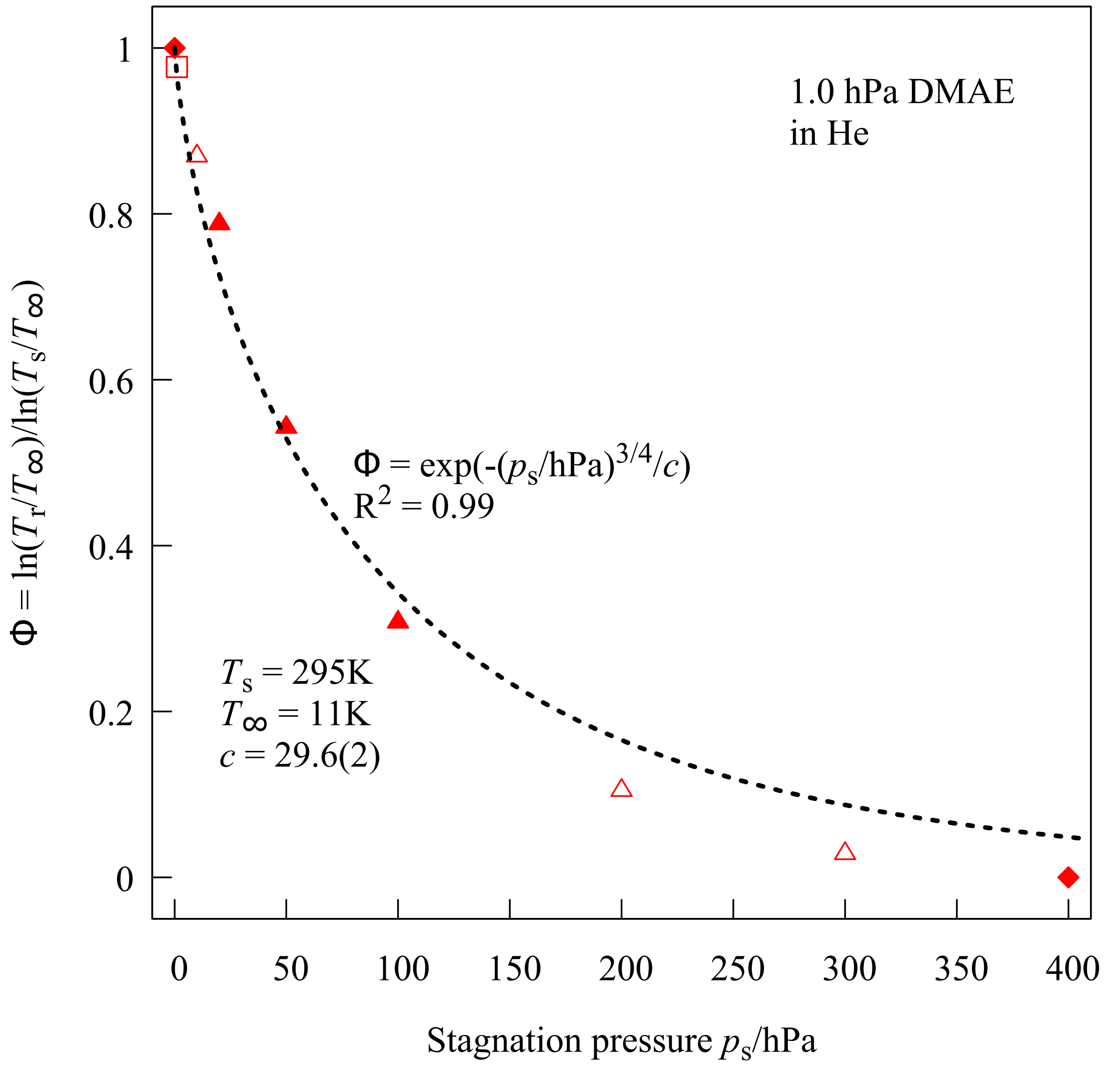

Figure S9: Rotational temperature function $\Phi(T_r)$ fitting for 1 hPa dimethylaminoethanol (DMAE) with He as carrier gas. Filled symbols are included in the fit, empty symbols are test data. The diamond symbols mark measurements at $T = T_s$ and T_∞, the squared symbol denotes a soft expansion without carrier gas, and all other data is marked with a triangle. The corresponding spectral series for all data points are shown in the Fig. S2.

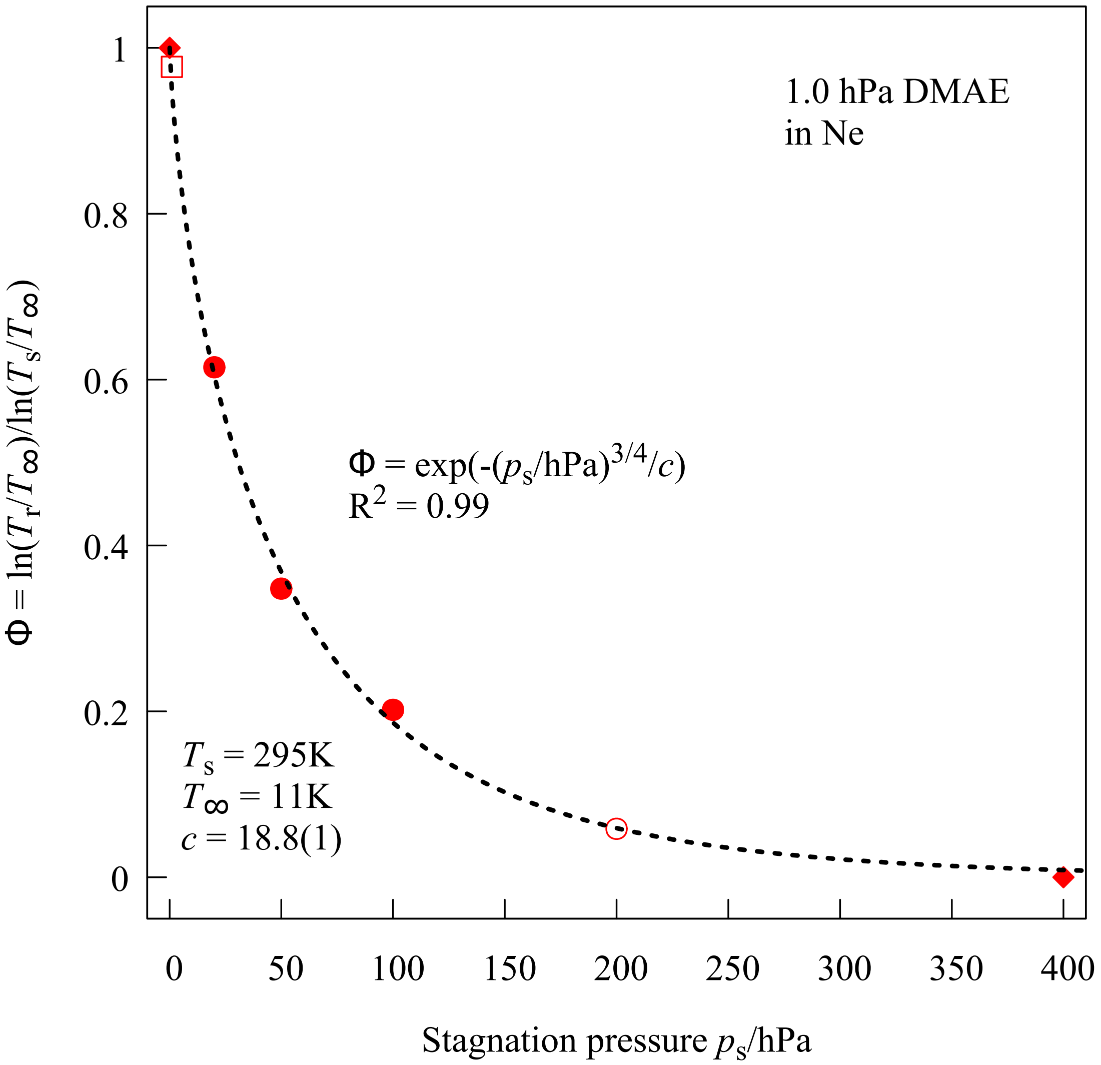

Figure S10: Rotational temperature function $\Phi(T_r)$ fitting for 1 hPa dimethylaminoethanol (DMAE) with Ne as carrier gas. Filled symbols are included in the fit, empty symbols are test data. The diamond symbols mark measurements at $T = T_s$ and T_∞, the squared symbol denotes a soft expansion without carrier gas, and all other data is marked with a circle. The corresponding spectral series for all data points are shown in the Fig. S3.

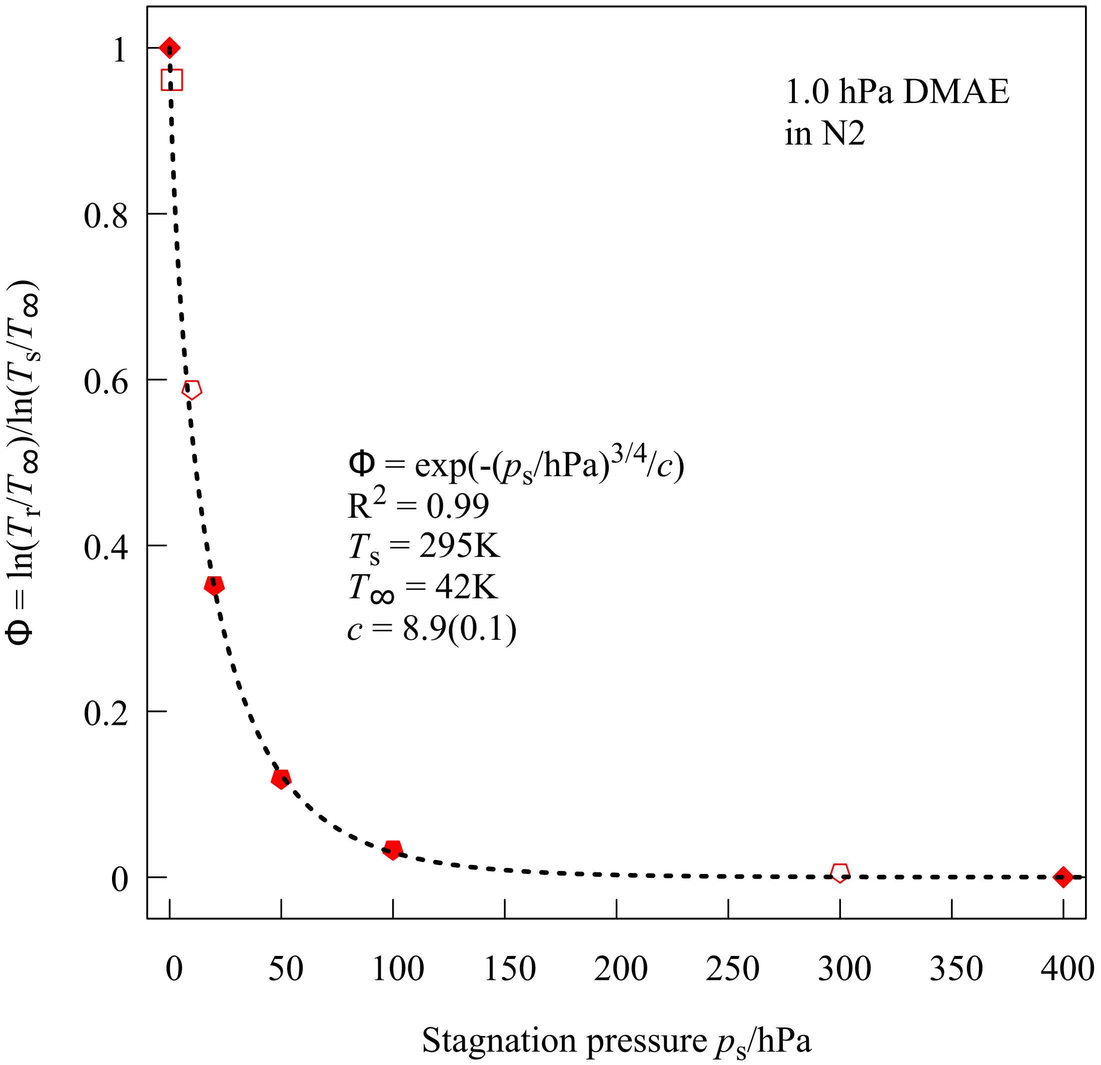

Figure S11: Rotational temperature function $\Phi(T_r)$ fitting for 1 hPa dimethylaminoethanol (DMAE) with N_2 as carrier gas. Filled symbols are included in the fit, empty symbols are test data. The diamond symbols mark measurements at $T = T_s$ and T_∞, the squared symbol denotes a soft expansion without carrier gas, and all other data is marked with a pentagon. The corresponding spectral series for all data points are shown in the Fig. S4.

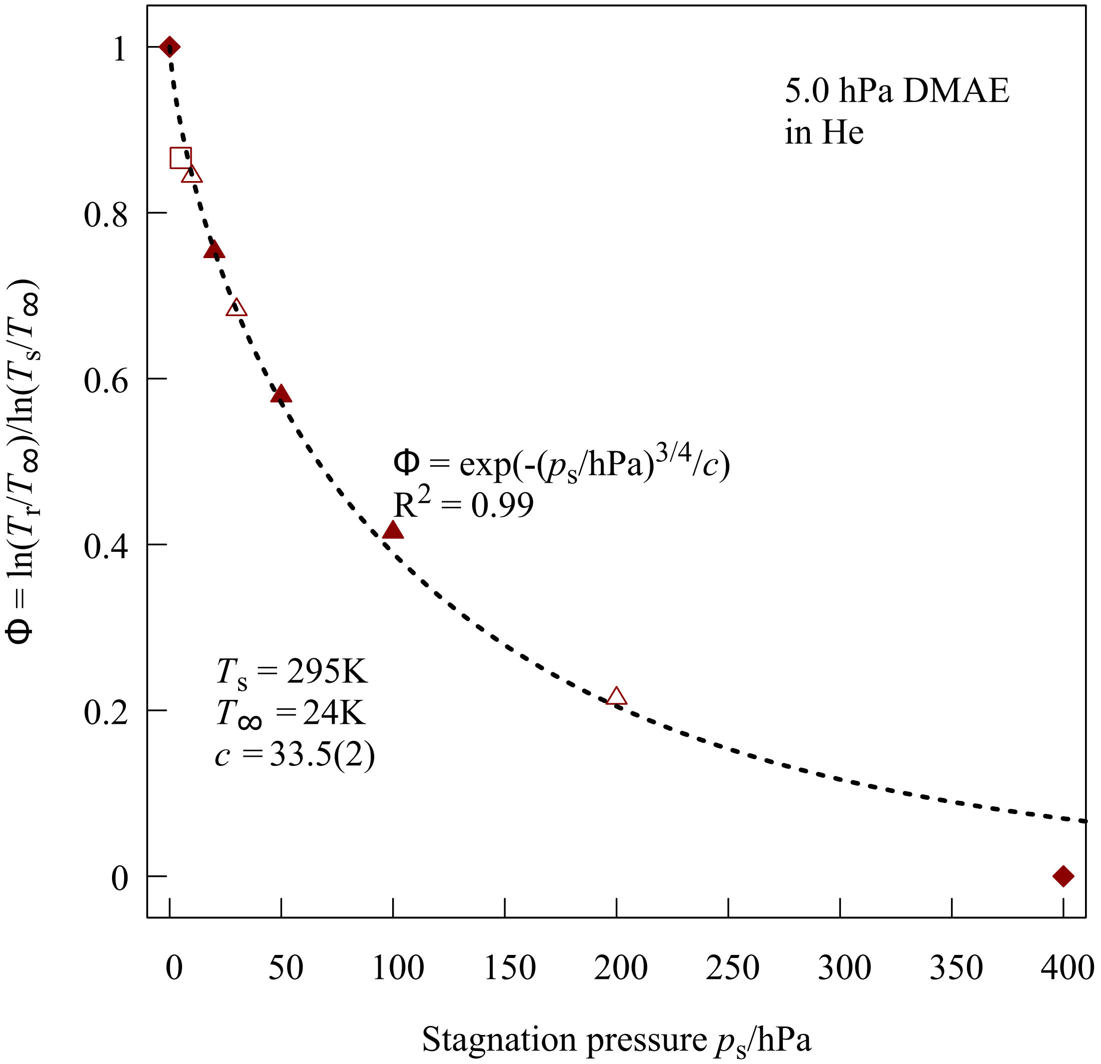

Figure S12: Rotational temperature function $\Phi(T_r)$ fitting for 5 hPa dimethylaminoethanol (DMAE) with He as carrier gas. Filled symbols are included in the fit, empty symbols are test data. The diamond symbols mark measurements at $T = T_s$ and T_∞, the squared symbol denotes a soft expansion without carrier gas, and all other data is marked with a triangle. The corresponding spectral series for all data points are shown in the Fig. S5.

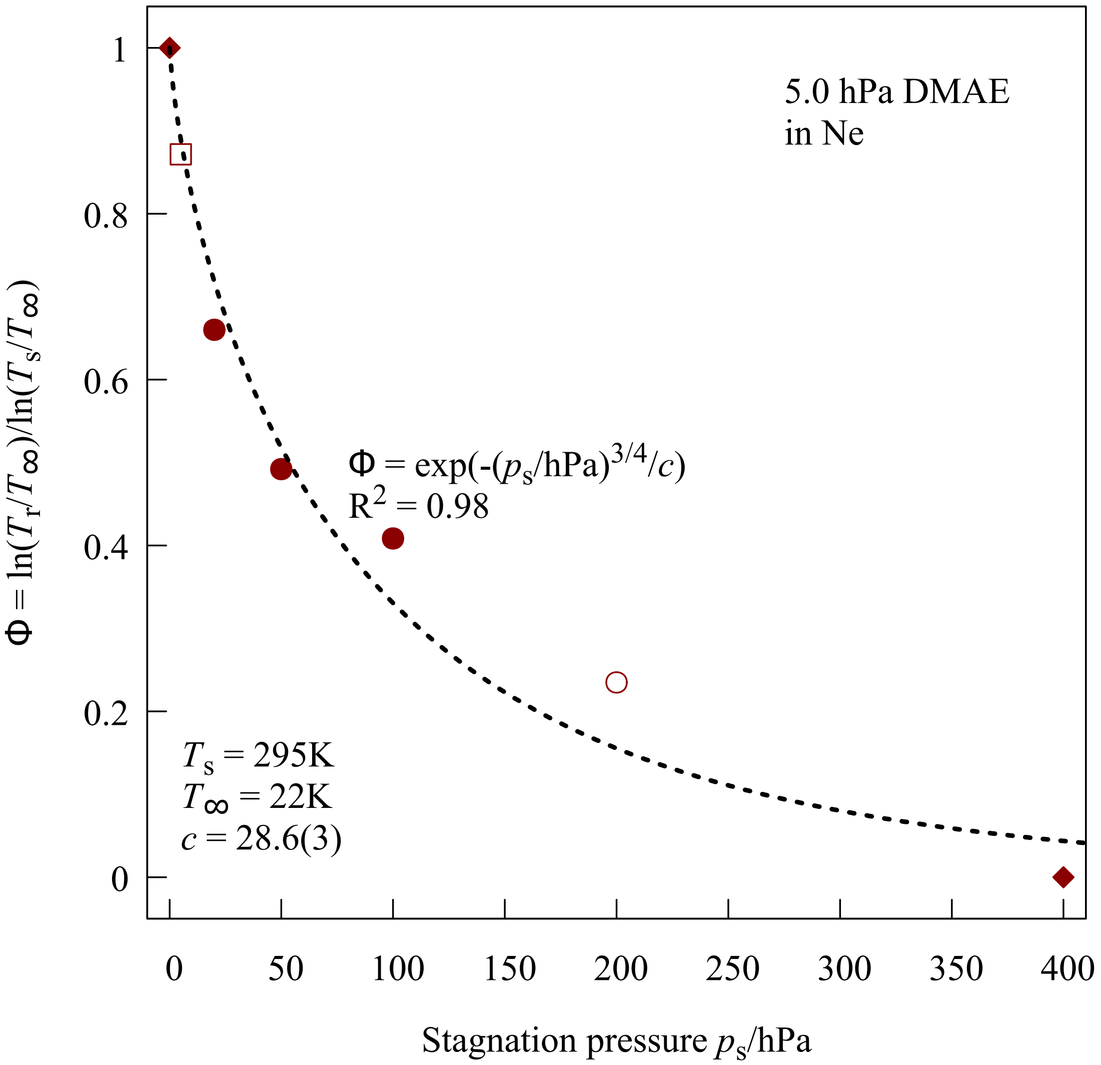

Figure S13: Rotational temperature function $\Phi(T_\mathrm{r})$ fitting for 5 hPa dimethylaminoethanol (DMAE) with Ne as carrier gas. Filled symbols are included in the fit, empty symbols are test data. The diamond symbols mark measurements at $T = T_\mathrm{s}$ and T_∞, the squared symbol denotes a soft expansion without carrier gas, and all other data is marked with a circle. The corresponding spectral series for all data points are shown in the Fig. S6.

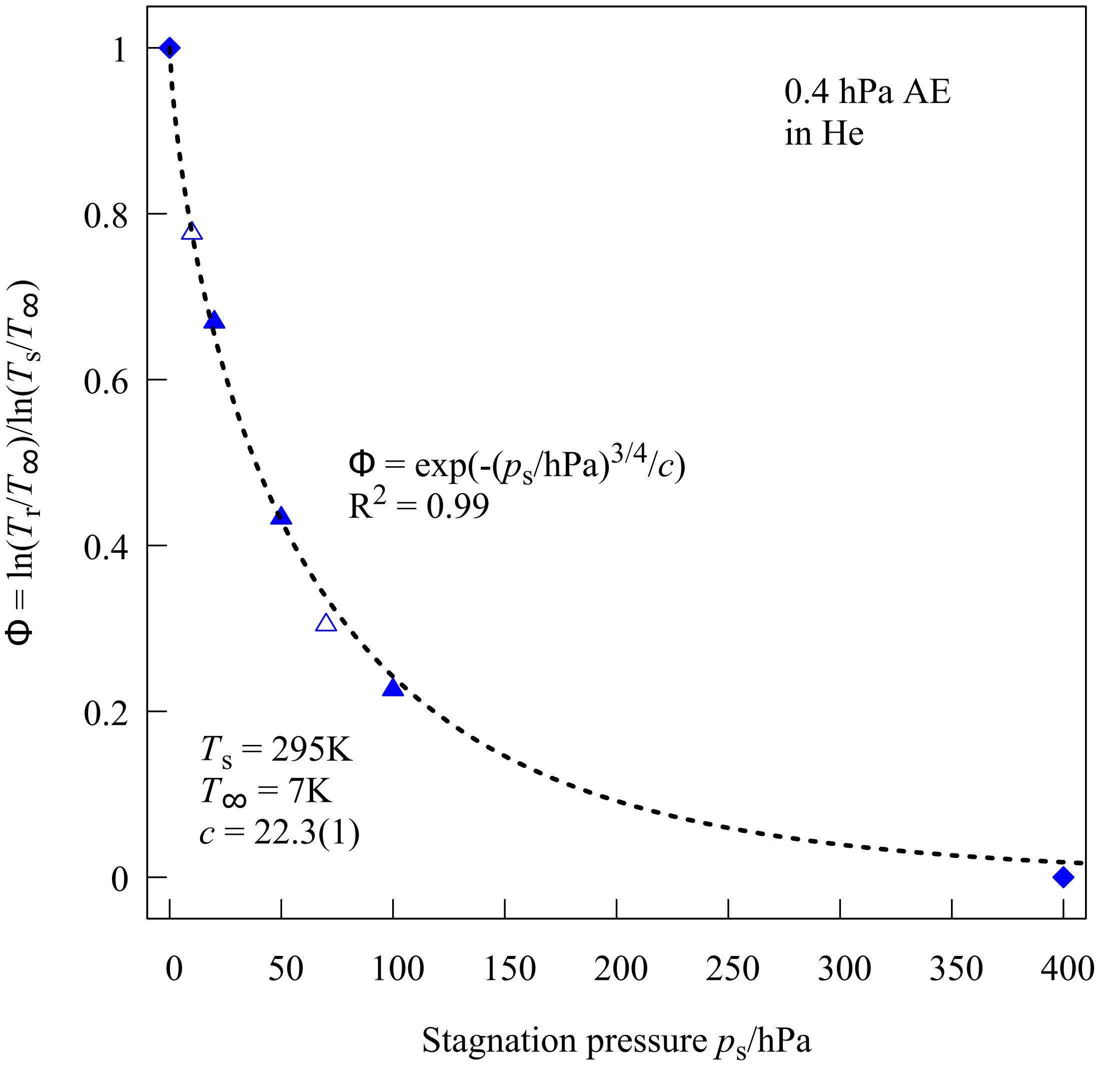

Figure S14: Rotational temperature function $\Phi(T_r)$ fitting for 0.4 hPa aminoethanol (AE) with He as carrier gas. Filled symbols are included in the fit, empty symbols are test data. The diamond symbols mark measurements at $T = T_s$ and T_∞, and all other data is marked with a triangle. The corresponding spectral series for all data points are shown in the Fig. S7.

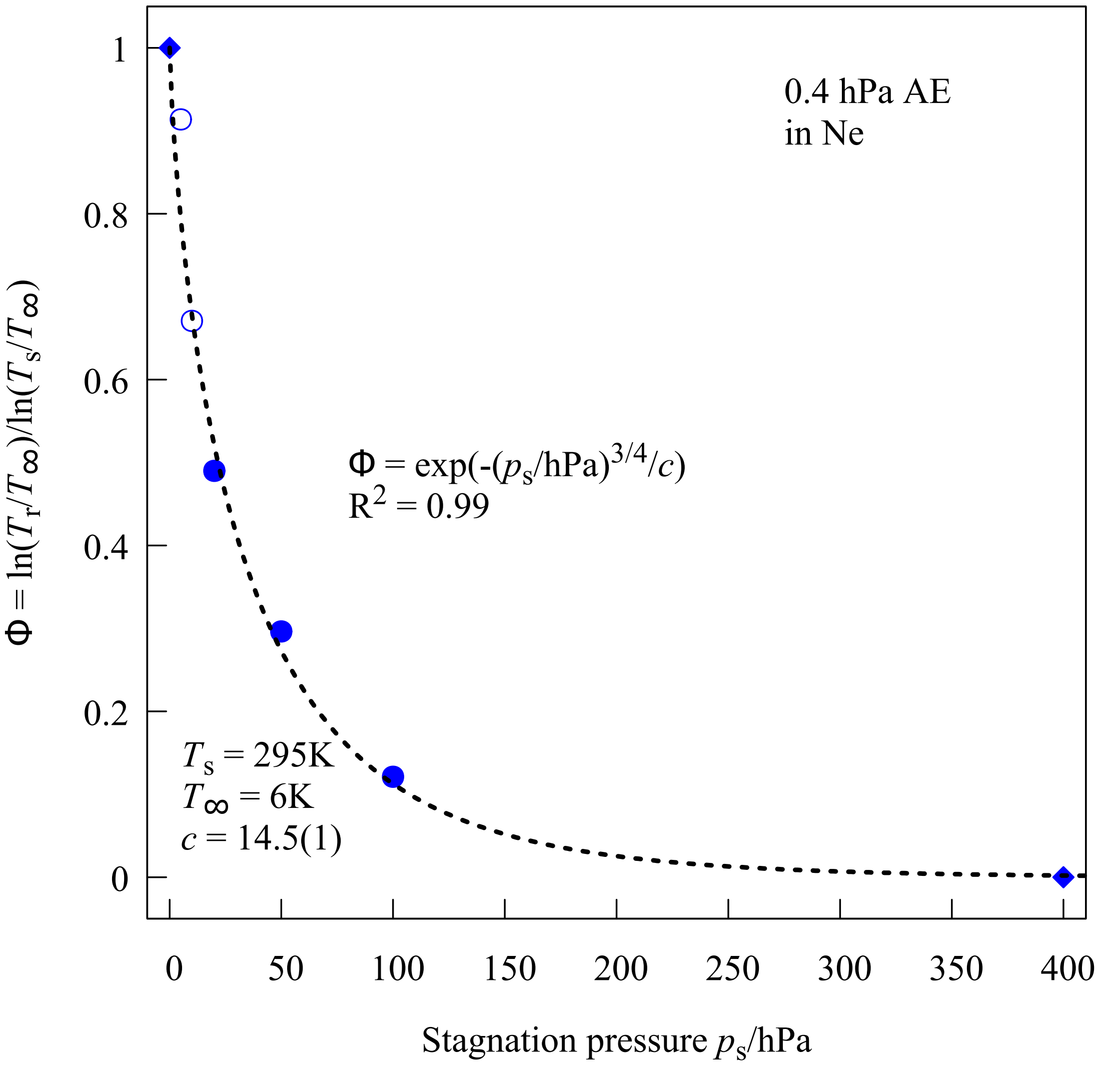

Figure S15: Rotational temperature function $\Phi(T_r)$ fitting for 0.4 hPa aminoethanol (AE) with Ne as carrier gas. Filled symbols are included in the fit, empty symbols are test data. The diamond symbols mark measurements at $T = T_s$ and T_∞, and all other data is marked with a circle. The corresponding spectral series for all data points are shown in the Fig. S8.

3 Vibrational temperatures

Vibrational temperatures in a supersonic jet are estimated using a Boltzmann population analysis approach. A simple, four-state model for visualisation of the relevant transitions is shown in figure S16.

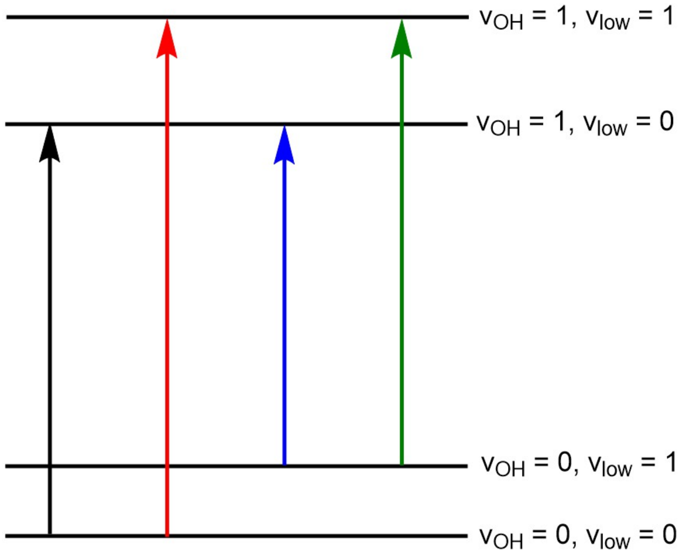

Figure S16: Schematic explanation of all transitions and states relevant for vibrational temperature estimations. Here, v_{OH} is the OH stretching vibration quantum number and v_{low} is the quantum number for a low frequency vibrational mode. The fundamental OH stretching transition (black), the sum transition (red), the difference transition (blue) and the hot transition (green) are shown.

Only those vibrations which strongly couple to the OH stretching vibration give rise to significant sum and difference bands. The latter are furthermore only visible if the vibrational temperature is sufficiently high. Therefore, vibrational temperatures can only be obtained for specific vibrations and soft expansions. We compare the experimental intensity of a difference band, where the low frequency vibrational mode of interest is already in the first excited state, to the intensity of the fundamental band or the sum band, where it is not. All bands include hot transitions with respect to other low frequency modes, which are expected to roughly cancel for relative intensities. The intensity of the fundamental band also includes the first hot band of the mode of interest, which may lead to a systematic overestimate of its intensity. The alternative use of the sum band has the disadvantage of a lower intensity, introducing a larger statistical error. In the case of DMAE, overlap with water monomer bands can also be a problem. Therefore, the intensity ratio between the difference and fundamental band gives more precise, but not necessarily more accurate results.

The vibrational temperature of a low frequency vibrational mode is modelled using equation S2.

$$T_\mathrm{v} = \frac{hc\Delta\tilde{\nu}}{k \ln\left(\frac{I_2 C}{I_1}\right)} \tag{S2}$$

Here, T_v is the vibrational temperature, h is Planck's constant, c is the speed of light, $\Delta\tilde{\nu}$ is the photon wavenumber needed for the excitation of the first low frequency vibrational mode quantum, k is the Boltzmann constant, I_1 is the experimental intensity of the difference transition, I_2 is the experimental intensity of the fundamental or sum transition and C is a calibration constant. The calibration constant needed for vibrational temperature estimation requires a measurement where the vibrational temperature is known. Thus, a laboratory temperature spectrum where vibrational temperatures equal the laboratory temperature is measured, and the calibration constant is obtained using equation S3.

$$C = \frac{I_1}{I_2} \cdot \exp\left(\frac{hc\Delta\tilde{\nu}}{kT_\mathrm{v}}\right). \tag{S3}$$

A sample spectrum of dimethylaminopropanol (DMAP) that presents two sum and two difference bands is shown in Fig. S17. The difference band closer to the fundamental band matches the harmonic wagging prediction of the dimethylamino group (Wag), while the difference band that is further away matches the harmonic ON stretching vibration of the intramolecular hydrogen bond of DMAP.
Under cryogenic conditions, there is little to no thermal excitation to the excited state of the low frequency vibrational mode. The difference bands of the jet cooled spectrum in Fig. S17 vanish due to the lack of excited state molecules that show difference transitions. So, using this method, one can only estimate the vibrational temperatures as long as the difference band is distinguishable from noise.

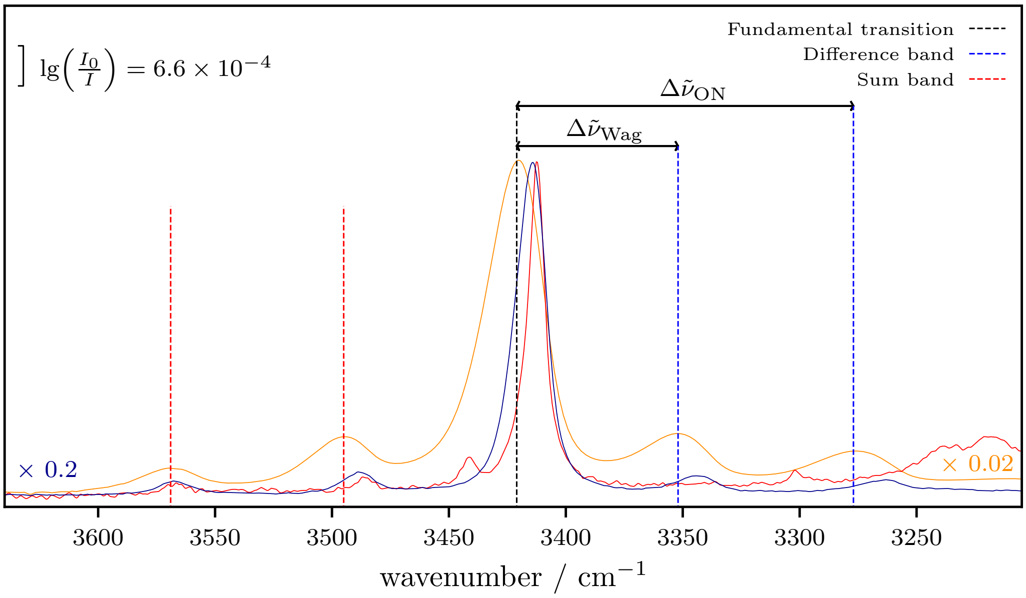

Figure S17: FTIR spectra of DMAP measured with the *gratin*-jet spectrometer at laboratory temperature (orange), softly expanded using 1 hPa of DMAP in 49 hPa of helium (blue) and jet cooled using 0.2 hPa of DMAP in 400 hPa of neon. The experimental parameters relevant to the estimation of vibrational temperatures are labelled in the spectrum. Spectral assignments for the DMAP signals are shown in Fig. S27.

3.1 Integration method

To calculate the vibrational temperature T_v of the low frequency modes Wag and ON which are coupling to the fundamental OH stretching vibration, the fundamental OH band, sum bands (OH + Wag, OH + ON) and difference bands (OH - Wag, OH - ON) are integrated. The spectra in the range 3200 - 3700 cm^{-1} as shown in Fig. 6 of the main text are evaluated to obtain the intensities I of individual bands with their uncertainty ΔI. The band center $\tilde{\nu}$ weighted over the intensity is the center of gravity of the band with its uncertainty $\Delta\tilde{\nu}$.
The intensity I and the band center $\tilde{\nu}$ are estimated by using the integration method which is largely based on the NoisySignalIntegration method by Nils O. B. Lüttschwager [8]. It allows for different settings of the integration windows and also provides a local baseline that is evaluated based on the signal to noise. In brief, the program analyses the actual noise in the selected wavenumber range of the spectrum and uses this to simulate additional noise with the same characteristics to the spectrum before the integration is performed. For the soft expansion spectra analysed in Table S7, spectral noise is not the limiting factor due to the uncertainty of the overlapping band boundaries. Therefore, in this case a uniform uncertainty of 5% is applied to the integrated area to estimate the uncertainty of vibrational temperature ΔT_v.
To perform the integration, a more or less symmetric integration window around the band maximum is chosen to cover the whole signal above the noise level. The integration boundaries are allowed to vary by 1 cm^{-1}. To give an example for the OH band of the soft expansion spectrum (1.5 hPa dimethylaminopropanol (DMAP) with 8.5 hPa helium (He)), the low integration boundary is sampled in the 3381-3382 cm^{-1} range and the high integration boundary in the 3463-3464 cm^{-1} range.
The integrated intensities I, the band center $\tilde{\nu}$ and their uncertainties ΔI and $\Delta\tilde{\nu}$ are tabulated in Table S7 for dimethylaminoethanol (DMAE) and in Table S8 for dimethylaminopropanol (DMAP).

Table S7: Experimental band centers $\tilde{\nu}$ and their uncertainties $\Delta\tilde{\nu}$ as well as IR intensities I and their uncertainties ΔI for the fundamental OH band, sum band (OH + ON) and difference band (OH - ON) of dimethylaminoethanol (DMAE).

Band	$\tilde{\nu}$	$\Delta\tilde{\nu}$	I	ΔI
DMAE laboratory temperature				
OH	3549.11	0.00	1.4002	6.3E-05
OH+ON	3670.67	0.02	0.0636	4.1E-05
OH-ON	3416.17	0.00	0.1916	4.5E-05
1 hPa DMAE + 19 hPa He				
OH	3544.15	0.04	0.0090	4.5E-04
OH+ON	3676.38	0.71	0.0004	1.9E-05
OH-ON	3413.62	0.25	0.0007	3.3E-05
5 hPa DMAE + 15 hPa He				
OH	3544.91	0.02	0.0406	2.0E-03
OH+ON	3672.39	0.21	0.0008	3.8E-05
OH-ON	3413.07	0.09	0.0026	1.3E-04
1 hPa DMAE + 19 hPa Ne				
OH	3542.88	0.03	0.0080	4.0E-04
OH+ON	3669.34	0.44	0.0002	1.1E-05
OH-ON	3412.04	0.24	0.0004	1.9E-05

The hot band contributions to the fundamental band profile can also introduce a systematic error for the intensities of sum bands in dimethylaminopropanol (DMAP). The intensities of sum bands are corrected by subtracting the areas which are believed to be overlapped by hot band contributions to the fundamental region. The subtracted area (subtracted I) is calculated as one half of the ratio of S_M (sum band minimum which is connected with fundamental OH) over S_P (sum band peak) times the integrated sum band area I. The S_M, S_P, subtracted I and corrected I for the sum bands of DMAP are shown in Table S9.

Table S8: Experimental band center $\tilde{\nu}$ and their uncertainties $\Delta\tilde{\nu}$ as well as IR intensities I and their uncertainties ΔI of fundamental OH band, two sum bands: one for higher frequency mode (OH + ON) and one for lower frequency mode (OH + Wag) and two difference bands: (OH - ON) and (OH - Wag) of dimethylaminopropanol (DMAP).

Band	$\tilde{\nu}$	$\Delta\tilde{\nu}$	I	ΔI
DMAP laboratory temperature				
OH	3423.34	0.00	0.7433	5.7E-05
OH+ON	3570.79	0.01	0.0396	4.0E-05
OH-ON	3277.52	0.01	0.0737	4.8E-05
OH+Wag	3496.95	0.01	0.0669	3.3E-05
OH-Wag	3352.78	0.01	0.0789	4.3E-05
1.5 hPa DMAP + 8.5 hPa He				
OH	3420.29	0.03	0.0280	1.4E-03
OH+ON	3567.48	0.27	0.0016	8.2E-05
OH-ON	3272.84	0.17	0.0021	1.0E-04
OH+Wag	3495.66	0.13	0.0023	1.2E-04
OH-Wag	3350.35	0.10	0.0024	1.2E-04
1.5 hPa DMAP + 18.5 hPa He				
OH	3418.71	0.01	0.0402	2.0E-03
OH+ON	3568.23	0.11	0.0017	8.4E-05
OH-ON	3270.01	0.11	0.0020	1.0E-04
OH+Wag	3492.67	0.08	0.0027	1.3E-04
OH-Wag	3348.93	0.08	0.0027	1.4E-04

Table S9: Experimental S_M, S_P, one half of the ratio, subtracted intensity I and corrected intensity I for the sum bands (OH + ON, OH + Wag) of dimethylaminopropanol (DMAP) in the gas phase and soft expansion expansion spectra.

Band	S_M	S_P	$(S_M/S_P)/2$	subtracted I	corrected I
DMAP laboratory temperature					
OH+ON	3.86E-05	9.45E-05	0.2042	0.0081	0.0315
OH+Wag	1.07E-04	2.06E-04	0.2604	0.0174	0.0495
1.5 hPa DMAP + 8.5 hPa He					
OH+ON	1.72E-05	8.16E-05	0.1054	0.0002	0.0015
OH+Wag	5.58E-05	1.42E-04	0.1969	0.0005	0.0019
1.5 hPa DMAP + 18.5 hPa He					
OH+ON	8.60E-06	6.01E-05	0.0715	0.0001	0.0016
OH+Wag	3.86E-05	1.12E-04	0.1728	0.0005	0.0022

3.2 Vibrational temperature of ON and Wag

Table S10: Vibrational temperature T_v of ON in dimethylaminoethanol (DMAE). The calibration constant C, experimental $\Delta\tilde{\nu}_\mathrm{ON}$, experimental fundamental transition intensity I_OH and difference transition intensity $I_\mathrm{OH\text{-}ON}$ and the calculated vibrational temperature using equation S2 with its uncertainty are shown.

Condition	C	$\Delta\tilde{\nu}_\mathrm{ON}/\mathrm{m}^{-1}$	I_OH	$I_\mathrm{OH\text{-}ON}$	T_v /K	ΔT_v/K
1 hPa DMAE + 19 hPa He	0.26048	13200	0.0090	0.0007	148	12
5 hPa DMAE + 15 hPa He	0.26048	13200	0.0406	0.0026	136	10
1 hPa DMAE + 19 hPa Ne	0.26048	13200	0.0080	0.0004	111	7

Table S11: Vibrational temperature T_v of ON in dimethylaminopropanol (DMAP). The calibration constant C, experimental $\Delta\tilde{\nu}_\mathrm{ON}$, experimental fundamental transition intensity I_OH, sum transition intensity $I_\mathrm{OH+ON}$ and difference transition intensity $I_\mathrm{OH\text{-}ON}$ and the calculated vibrational temperature for fundamental method and sum band method using S2 with its uncertainty are shown.

Condition	C	$\Delta\tilde{\nu}_\mathrm{ON}/\mathrm{m}^{-1}$	I_OH	$I_\mathrm{OH\text{-}ON}$	T_v /K	ΔT_v/K
1.5 hPa DMAP + 8.5 hPa He	0.2011	14500	0.0280	0.0021	207	21
1.5 hPa DMAP + 18.5 hPa He	0.2011	14500	0.0402	0.0020	149	11
Condition	C	$\Delta\tilde{\nu}_\mathrm{ON}/\mathrm{m}^{-1}$	$I_\mathrm{OH+ON}$	$I_\mathrm{OH\text{-}ON}$	T_v /K	ΔT_v/K
1.5 hPa DMAP + 8.5 hPa He	4.7420	14500	0.0015	0.0021	171	15
1.5 hPa DMAP + 18.5 hPa He	4.7420	14500	0.0016	0.0020	159	13

Table S12: Vibrational temperature T_v of Wag in dimethylaminopropanol (DMAP). The calibration constant C, experimental $\Delta\tilde{\nu}_\mathrm{Wag}$, experimental fundamental transition intensity I_OH, sum transition intensity $I_\mathrm{OH+Wag}$ and difference transition intensity $I_\mathrm{OH\text{-}Wag}$ and the calculated vibrational temperature for fundamental method and sum band method using S2 with its uncertainty are shown.

Condition	C	$\Delta\tilde{\nu}_\mathrm{Wag}/\mathrm{m}^{-1}$	I_OH	$I_\mathrm{OH\text{-}Wag}$	T_v /K	ΔT_v/K
1.5 hPa DMAP + 8.5 hPa He	0.1478	6800	0.0280	0.0024	181	35
1.5 hPa DMAP + 18.5 hPa He	0.1478	6800	0.0402	0.0027	126	16
Condition	C	$\Delta\tilde{\nu}_\mathrm{Wag}/\mathrm{m}^{-1}$	$I_\mathrm{OH+Wag}$	$I_\mathrm{OH\text{-}Wag}$	T_v /K	ΔT_v/K
1.5 hPa DMAP + 8.5 hPa He	2.2198	6800	0.0019	0.0024	178	38
1.5 hPa DMAP + 18.5 hPa He	2.2198	6800	0.0022	0.0027	167	33

4 Modelling

4.1 Details on harmonic calculations

The molecular structures of aminoethanol (AE), methylaminoethanol (MAE), dimethylaminoethanol (DMAE) and its monohydrate (w-DMAE) as well as dimethylaminopropanol (DMAP) were optimised by using three-body-inclusive D3-dispersion-corrected[9,10] B3LYP with the def2-TZVP[11–13] basis set (B3LYP/TZ) and ma-def2-TZVP basis set[11–13] (B3LYP/maTZ) using the program ORCA 5.0.3[14–17] with the keywords ABC DEFGRID3 VERYTIGHTSCF VERYTIGHTOPT FREQ mass2016. The initial monomers and dimers were drawn in a chemically reasonable way using Chemcraft version 1.8. By using CREST[18,19], the possible monomers and dimers structures were simulated. The generated monomers and dimers were preoptimised at B3LYP-D3/def2-TZVP level on the program ORCA 5.0.3. After that, the lowest energy conformers were reoptimised again at B3LYP-D3/ma-def2-TZVP level.
For the 5 different DMAE dimers, higher level calculations using the B2PLYP[20] functional with ma-def2-TZVP and def2-QZVP basis sets were performed. The electronic energy of DMAE dimers was also optimised at DLPNO-CCSD(T)[21] level using the "TightPNO" settings with the aug-cc-pVQZ[22–24] basis set at the optimised B3LYP and B2PLYP geometries, to get the relative energy difference as shown in Table S16 and S17. The single point DLPNO-CCSD(T) calculations were performed by using the keywords DLPNO-CCSD(T) TightPNO VeryTightSCF RIJK NoTRAH KDIIS Basis "aug-cc-pVQZ" AuxJK "aug-cc-pVQZ/JK" AuxC "aug-cc-pVQZ/C".
Similarly, the trans (t) and gauche (g) MAE monomers were optimised at B2PLYP/def2-QZVP level with ORCA 5.0.3. The relative electronic energy difference of trans (t) and gauche (g) was calculated by using DLPNO-CCSD(T), as described in Table S22.
The resulting optimised structures of monomers and dimers at B3LYP-D3/ma-def2-TZVP level with ORCA 5.0.3 are provided here: dataset.

4.2 Example inputs for NEB scans

An NEB scan[25] is used to calculate the energy barrier between two MAE conformers: trans (t) and gauche (g). The structure of the transition state from the NEB scan is reoptimised again by using B3LYP-D3/def2-TZVP on ORCA 5.0.3 with the keywords shown in Table S14. The figure for the energy barrier of MAE conformers: trans (t) and gauche (g), is shown in Fig.S25 of the supplementary information.
For convenience, example inputs for the NEB scan and transition state calculation are given in Table S13 and S14.

Table S13: Example input for NEB scan in the ORCA 5.0.3 calculations at the B3LYP level of computation. In the input file, "start.xyz" is the name of the beginning conformer and "end.xyz" is the name of the ending conformer that needed to convert.[25]

type of calculation	input
NEB scan	!NEB-CI B3LYP D3BJ abc def2-TZVP VeryTightSCF defgrid3 Mass2016<br>%neb<br>NEB_End_XYZFile "start.xyz"<br>SpringType DOF<br>PerpSpring cosTan<br>Tol_MaxF_CI 2.e-3<br>Tol_RMSF_CI 1.e-3<br>Tol_Scale 10.0<br>Local true<br>Nimages 30<br>end<br>%pal nprocs 30 end<br>%Maxcore 3000<br>*xyzfile 0 1 end.xyz

Table S14: Example input for the transition state calculation in the ORCA 5.0.3 calculations at the B3LYP level of computation.[26]

type of calculation	input
Transition state	!B3LYP D3BJ abc def2-TZVP UseSym SlowConv OptTS VeryTightOpt<br>VeryTightSCF Freq defgrid3 Mass2016<br>%method SymThresh 5.0e-2 end<br>%geom<br>Calc_Hess true<br>Recalc_Hess 3<br>ENFORCESTRICTCONVERGENCE true<br>MaxIter 500<br>end<br>%pal nprocs NUMCORES end<br>%Maxcore 3000<br>*xyzfile 0 1 Start.xyz

5 Spectral assignments

5.1 Nomenclature

For the assignments of the bands found in the spectra of the compounds studied in this work, a specific nomenclature for vibrational modes is used. The involved atoms are defined by capital letters; assignments are restricted to OH and NH stretching vibrations, which are labeled as "OH" or "NH". Monomers (M) and dimers (D) are distinguished by using a capital "M" or "D" in the superscript. In the subscript, differentiation between symmetric (sym) and asymmetric (asym) combinations of equivalent modes using "sym" or "asym" is made, where necessary.
Three different hydrogen bond topologies are found to be relevant in the investigated dimers and are visualized in Fig. S18. Insertion (i) and head-to-tail or mutually hydrogen-bonded (m) amino alcohol dimers are marked using

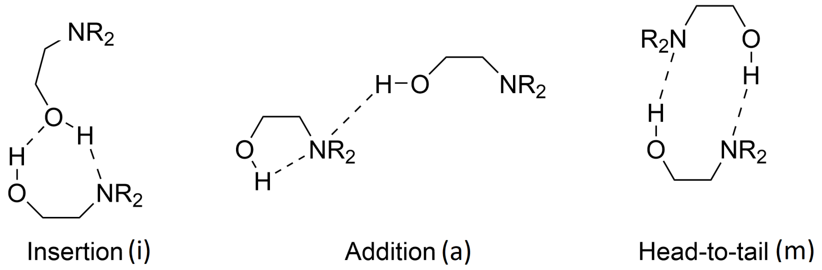

Figure S18: Possibilities for the intermolecular interactions in amino alcohol dimers studied in this work, modified from reference [27].

a subscript "i" or "m". The addition topology could not be assigned unambiguously in the experimental spectra of amino alcohol dimers, but has been discussed for monohydrates.[28] The transient chirality of aminoethanols with respect to the NCCO dihedral angle leads to homochiral (hom, same sign of the dihedral) and heterochiral (het, opposite sign of the dihedral) stereoisomers. The dihedral angle is visualized in Fig. S19 using DMAE as an example. For DMAP, a modified nomenclature would be required, but is not relevant in this work. A summary of all aspects of

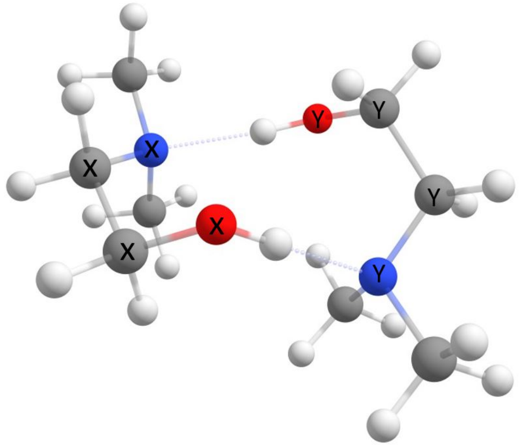

Figure S19: Dihedral angles of an exemplary dimethylaminoethanol dimer that determine the relative chirality label. The atoms included in the dihedral angle are labelled "X" for one dimethylaminoethanol unit and "Y" for the other unit. The structure shown is the heterochiral head-to-tail structure m_{hetA} of dimethylaminoethanol (DMAE).

the nomenclature is found in Table S15.
For computed structures, a simplified version of the spectral assignment nomenclature explained above is used. Monomers are labelled "M", while dimers are distinguished using "m" for head-to-tail structures and "i" for insertion structures. For dimers, the chirality is now defined using a subscript "hom" or "het". Variants are denoted with capital letters (A, B). The DMAE dimer case is summarised in Figure S20. Any exceptions to this nomenclature will be specified in the text.

5.2 Dimethylaminoethanol (DMAE)

The dimers of DMAE can realise insertion and head-to-tail topologies in both homochiral and heterochiral variants and thus provide an opportunity to test different theory levels with respect to their ability to reproduce the experimental spectral patterns. This is a prerequisite for the interpretation of conformational relaxation studies.

Table S15: Nomenclature used for the spectral features observed in this work.

object / feature	code	code position
vibrating atoms	atom abbreviation	capital letter
monomer	”M”	superscript
dimer	”D”	superscript
cluster	”C”	capital letter
asymmetric vibration (out-of-phase)	”asym”	subscript
symmetric vibration (in phase)	”sym”	subscript
insertion structure	”i”	subscript
head-to-tail structure	”m”	subscript
heterochirality	”het”	superscript
homochirality	”hom”	superscript

As discussed in the main text, an interesting computational ambiguity refers to the most stable head-to-tail heterochiral dimer (m_{het}), whereas the homochiral dimer is predicted uniformly. Variant A of the het dimer is predicted $2\,kJ\,mol^{-1}$ more stable at B3LYP level than variant B, but this systematically inverts at higher electronic structure level. The calculations in the main text use the def2-QZVP basis set (Table S16). Variations induced by switching to the ma-def2-TZVP basis set (Table S17) are small, as further illustrated in the analogous Fig. S21.

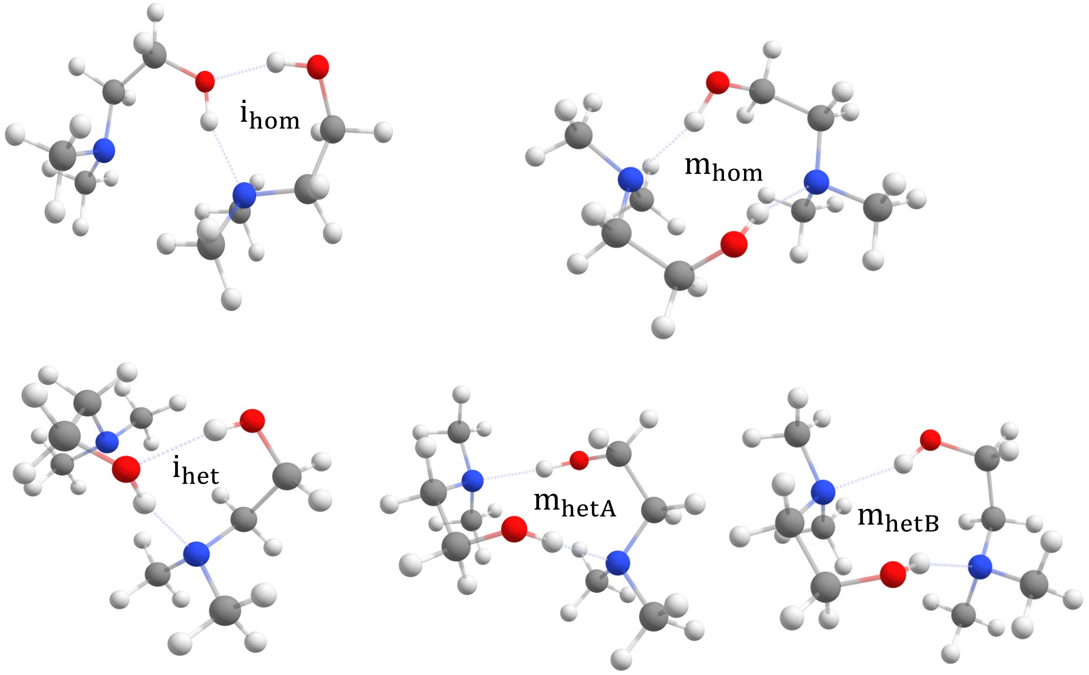

Figure S20: Structures of the most stable conformers of the DMAE dimer, optimised at B3LYP/def2-TZVP level.

Table S16: Relative zero point corrected ΔE_0 energy for 5 different conformers of dimethylaminoethanol dimers using the def2-QZVP basis set. These conformer structures are shown in Fig. S20

Conformer	B3LYP@B3LYP	B2PLYP@B2PLYP	DLPNO-CCSD(T)@B3LYP	DLPNO-CCSD(T)@B2PLYP
m_{hom}	0.00	0.00	0.00	0.00
m_{hetA}	0.32	0.57	0.92	1.32
m_{hetB}	2.52	1.71	0.88	0.77
i_{hom}	2.26	2.07	2.35	2.77
i_{het}	0.07	0.16	0.61	0.89

Table S17: Relative zero point corrected ΔE_0 energy for 5 different conformers of dimethylaminoethanol dimers using the ma-def2-TZVP basis set.

Conformer	B3LYP@B3LYP	B2PLYP@B2PLYP	DLPNO-CCSD(T)@B3LYP	DLPNO-CCSD(T)@B2PLYP
m_{hom}	0.00	0.00	0.00	0.00
m_{hetA}	0.13	0.27	0.95	1.37
m_{hetB}	2.48	1.57	0.79	0.77
i_{hom}	2.03	1.87	2.45	2.62
i_{het}	0.01	0.08	0.67	0.72

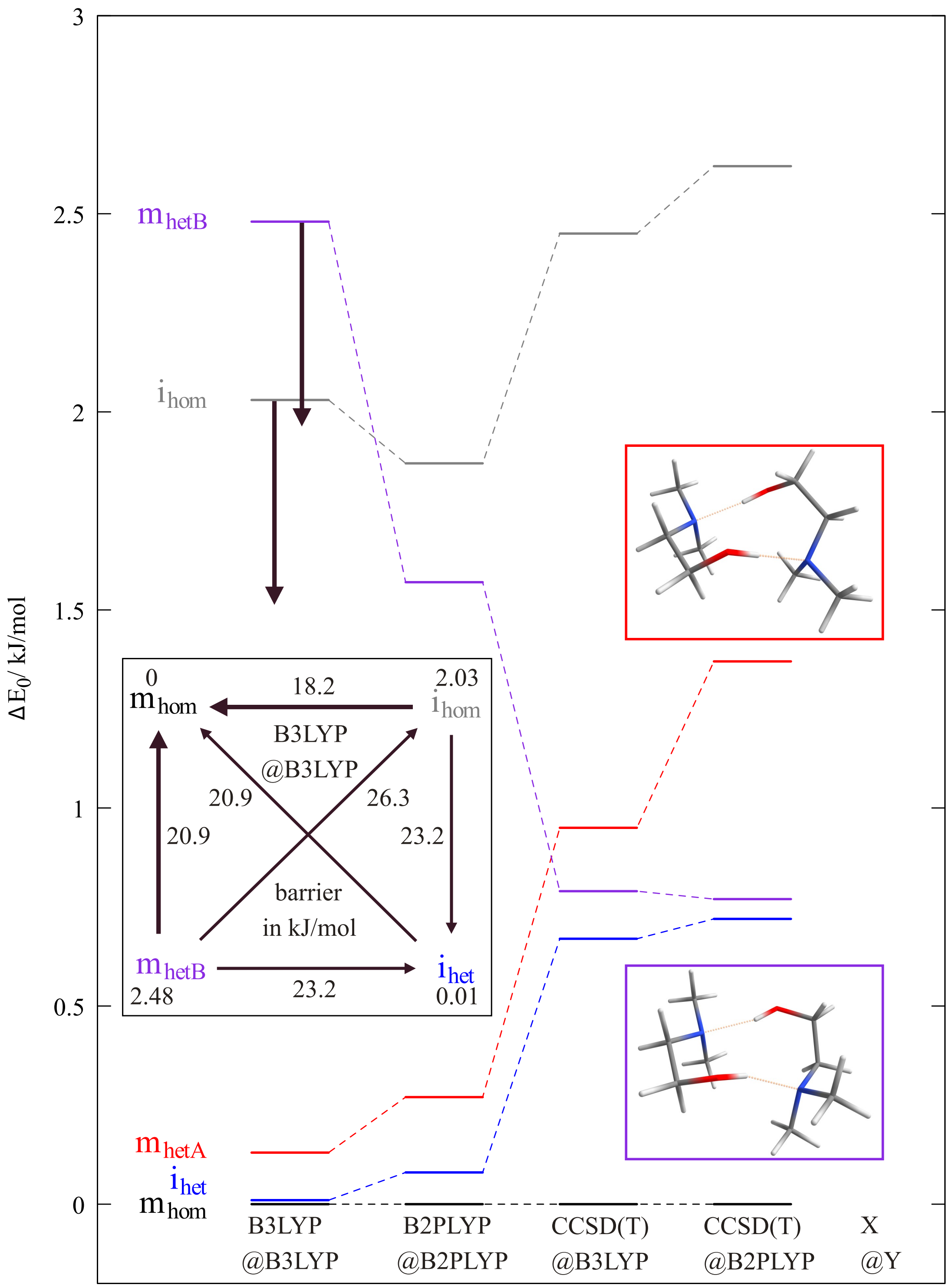

Figure S21: Relative energy of the hom(ochiral) and het(erochiral) dimers of DMAE as a function of electronic structure level X@Y (electronic energy at X level after structure optimization at Y level, harmonic zero point energy added at Y level; X=CCSD(T) stands for DLPNO-CCSD(T)) using the ma-def2-TZVP basis set. Insertion (i) and mutual OHN hydrogen bonding (m) compete with each other. The relative energy of the two depicted m_{het} isomers depends strongly on the theory level and can serve as an experimental benchmark because they differ spectroscopically. The possible pathways for relaxation are depicted as an insert with zero-point-corrected barriers at B3LYP level. Thick arrows denote the most facile interconversions.

Raman and FTIR spectra of DMAE and its dimers are shown in Fig. S22. Symmetric (in-phase) combinations typically have more intensity in the Raman spectra, asymmetric (out-of-phase) combinations in the FTIR spectra, even in the absence of inversion symmetry which would mutually exclude IR/Raman activity.

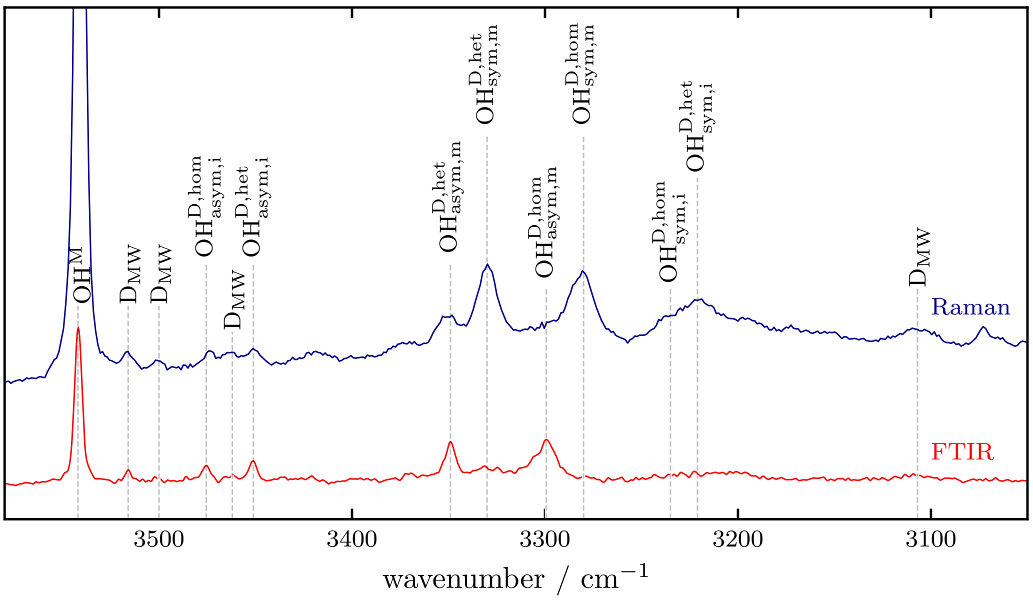

Figure S22: Raman and FTIR spectra of DMAE under cryogenic supersonic expansion conditions (see table S2 and table S4). The symmetric and asymmetric OH-stretching vibrations of the monomer and dimer are shown and labelled accordingly. The bands that originate from a complex between water and DMAE are labelled "D_{MW}". A detailed assignment of the DMAE monohydrate bands is given in Fig. 9 and 10 in the main manuscript.

For the inserted hydrogen bond topology, there is a large spectral difference between the inserted OH group (which binds to N and correlates with in phase or symmetric stretching) and the OH group which has to give up its N contact upon dimerisation (correlating with out-of-phase or asymmetric stretching). This splitting is more visible in the Raman spectrum, although the computed intensities would suggest sufficient intensities in the FTIR spectrum as well. The spectral splitting due to hom and het pairs is more subtle.

The vibrational signature of the head-to-tail (m) structures falls in between the OH stretching bands caused by the inserted (i) structures. There are large differences between variants A and B of the m_{het} dimer, which are summarised in Table S18. Structure A is predicted to be spectrally downshifted relative to the homochiral structure, whereas structure B is spectrally upshifted relative to the homochiral structure.

Table S18: Overview of the results for B3LYP-D3 calculations using the maTZ basis for DMAE. The table lists the relative electronic energy to the most stable conformer ΔE_{el}, the relative ZPVE-corrected energy ΔE_0, the harmonic OH-stretching frequency ω_{OH}, the IR-intensity I_{IR}, the computed Raman intensity I_{Raman} (without experimental corrections) and the experimental wavenumbers $\tilde{\nu}$ for both FTIR and Raman experiments.

	Theory								Experiment			
conformer	ΔE_{el} / kJ mol^{-1}	ΔE_0 / kJ mol^{-1}	ω_{OH} / cm^{-1}		I_{IR} / km mol^{-1}		I_{Raman} / Å^4 u^{-1}		$\tilde{\nu}_{FTIR}$ / cm^{-1}		$\tilde{\nu}_{Raman}$ / cm^{-1}	
			asym	*sym*	*asym*	*sym*	*asym*	*sym*	*asym*	*sym*	*asym*	*sym*
Monomer												
M	-	-	3690		73		59		3542		3542	
Dimers												
D_i^{hom}	3.1	2.0	3572	3259	651	815	109	191	3476	-	3474	3235
D_i^{het}	0.6	0.1	3556	3232	490	873	63	182	3451	-	3451	3221
D_m^{hom}	0.6	0	3411	3386	1720	98	28	372	3299	-	-	3280
$D_m^{het,A}$	0	0.1	3349	3275	1480	763	141	266	-	-	-	-
$D_m^{het,B}$	3.0	2.5	3453	3417	1040	478	125	281	3349	3330	3350	3330

The IR band in Fig. S22 at about 3300 cm^{-1} and its Raman counterpart at lower wavenumber are assigned to the homochiral head-to-tail structure. This assignment is based on the predicted C_2 symmetry of the structure and the strong intensity complementarity. The dominant dipole and polarisability changes happen perpendicular to the C_2

axis. This complementarity is partially lifted in both variants of the heterochiral dimer m_{het}. Such a pattern of partially complementary FTIR/Raman bands is found experimentally (Fig. S22) at higher wavenumber than m_{hom}, thus confirming structure B to be more stable and preferentially formed.

5.3 Aminoethanol (AE)

FTIR and Raman jet spectra of AE are shown in Fig. S23. Compared to the DMAE case, the FTIR spectrum conveys a simple message with one prominent M and one prominent D transition, as shown before.[29]

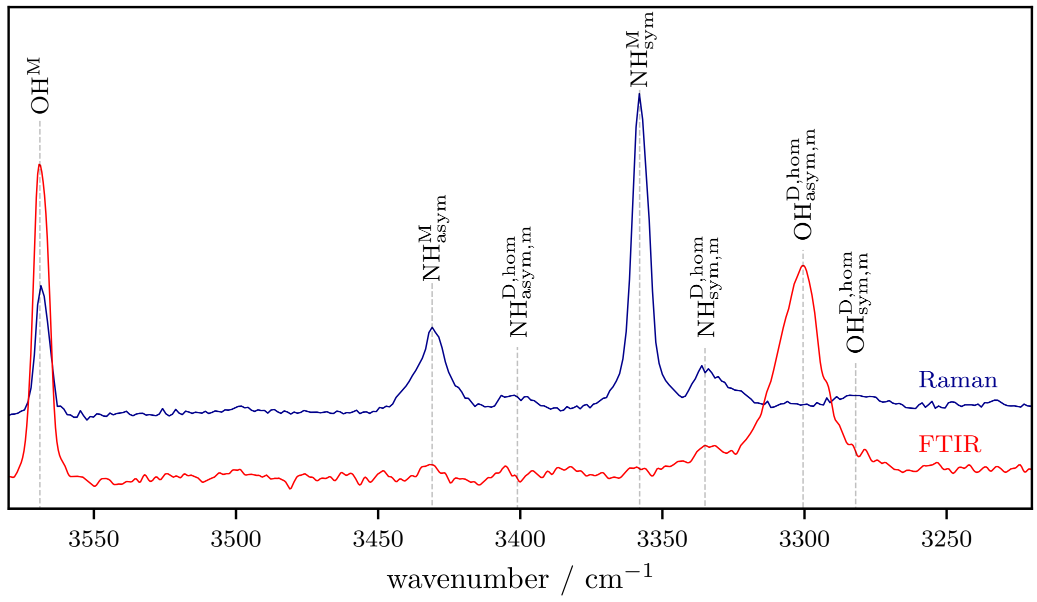

Figure S23: Raman and FTIR spectra of AE at cryogenic, supersonic expansion conditions. The symmetric and asymmetric OH- and NH-stretching vibrations of the monomer and dimer are shown and labelled accordingly.

The dimer signal is confirmed to match a homochiral head-to-tail structure, as proposed before.[27] The Raman spectrum shows some impurity and water evolution with gas recycling, presumably in part due to reactions in the hot multi-stage roots compression pump. It should therefore be interpreted cautiously. However, it amplifies NH-stretching vibrational modes of the monomer, which have a very low IR intensity and would not be assignable with the FTIR spectrum alone. The absence of NH bonds in DMAE is one of the reasons why the study of monohydrates (see main text) focusses on fully methylated N cases.

There are two very weak spectral contributions in the NH range of both the IR and the Raman spectra. Apart from impurity assignments, these could potentially be due to NH stretching modes in clusters, where the weak IR activity is partially lifted due to hydrogen bonding. For the transition at 3335 cm^{-1}, a plausible interpretation is harmonic mode-mixing with the nearby strong asymmetric dimer OH stretching vibration. Based on the intensity ratio and separation, a harmonic coupling matrix element of $\approx$5 cm^{-1} can be derived and this is close to 1/2 of the avoided crossing between asymmetric NH and OH modes when the mass of the hydrogen-bonded H atoms is tuned [30] between 1.0 and 0.9 to provoke mode mixing of a dark and a bright state. These assignments remain tentative. In the future, it will be rewarding to explore why AE dimers relax quantitatively to the most stable hydrogen bond topology and relative chirality, whereas DMAE dimers freeze into at least 4 conformational isomers.

Table S19: Overview of the OH results for B3LYP-D3 calculations using the maTZ basis set for AE and its homodimer. The table lists the harmonic OH-stretching frequency ω_{OH}, the IR-intensity I_{IR}, the Raman intensity I_{Raman} and the experimental OH-stretching wavenumbers $\tilde{\nu}$ for both FTIR and Raman experiments.

**OH**	Theory						Experiment			
conformer	ω_{OH} / cm^{-1}		I_{IR} / km mol^{-1}		I_{Raman} / Å^4 u^{-1}		$\tilde{\nu}_{\mathrm{FTIR}}$ / cm^{-1}		$\tilde{\nu}_{\mathrm{Raman}}$ / cm^{-1}	
	asym	*sym*	*asym*	*sym*	*asym*	*sym*	*asym*	*sym*	*asym*	*sym*
Monomer M	3703		62		52		3569		3569	
Dimer D_m^{hom}	3376	3346	1810	74	19	356	3300<br>3304[27]<br>≈3300[29]	-	-	3284

Table S20: Overview of the NH results for B3LYP-D3 calculations using the maTZ basis set for AE and its homodimer. The table lists the harmonic NH-stretching frequency ω_{NH}, the IR-intensity I_{IR}, the Raman intensity I_{Raman} and the experimental NH-stretching wavenumbers $\tilde{\nu}$ for both FTIR and Raman experiments.

**NH**	Theory						Experiment			
conformer	ω_{NH} / cm^{-1}		I_{IR} / km mol^{-1}		I_{Raman} / Å^4 u^{-1}		$\tilde{\nu}_{\mathrm{FTIR}}$ / cm^{-1}		$\tilde{\nu}_{\mathrm{Raman}}$ / cm^{-1}	
	asym	*sym*	*asym*	*sym*	*asym*	*sym*	*asym*	*sym*	*asym*	*sym*
Monomer M	3581	3498	5	0	65	123	3430[27]	-	3431	3358
Dimer D_m^{hom}	3548	3475	1	0	45	189	-	-	3401	3335
	3547	3475	13	5	63	6	-	3335?	3401	(3335)

5.4 Methylaminoethanol (MAE)

Raman and FTIR jet spectra of MAE in the monomer OH/NH stretching range are shown in Fig. S24.

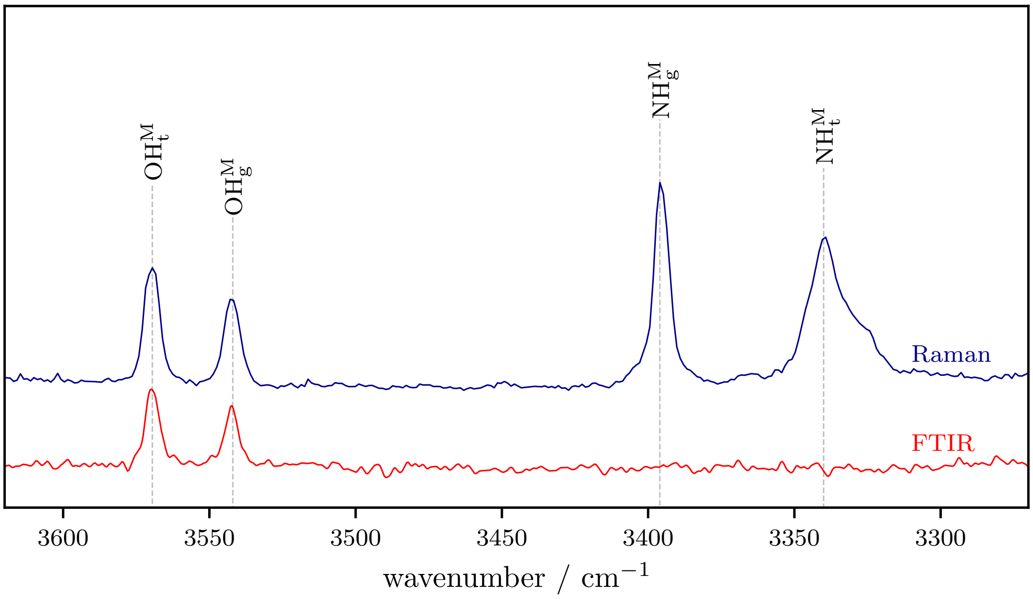

Figure S24: Raman and FTIR spectra of MAE at cryogenic, supersonic expansion conditions. The symmetric and asymmetric OH-stretching vibrations of both stable monomer configurations (trans (t) and gauche (g), labelled in the subscript of the nomenclature) are shown and labelled accordingly.

Harmonic predictions and experimental wavenumbers of MAE are listed in Table S21.

Table S21: Comparison between the results for the harmonic predictions at the B3LYP/maTZ level and the experimental results for MAE. The table lists the bands visible in the spectra, their experimental wavenumber $\tilde{\nu}_{exp}$ for both Raman and FTIR experiments where "-" means that this band is not seen in the experimental spectra, the scaled harmonic wavenumber predictions ω ($\omega_{theo} \times 0.96$), as well as the IR and Raman intensities $I_{IR,Raman}$. Spectral intensities were computed in the double-harmonic approximation.

vibrational	$\tilde{\nu}_{exp}$	/ cm^{-1}	ω	/ cm^{-1}	I_{IR}	/ $km\,mol^{-1}$	I_{Raman} / $Å^4\,u^{-1}$
mode	FTIR	Raman	B3LYP/maTZ	B2PLYP/QZ	B3LYP/maTZ	B2PLYP/QZ	B3LYP/maTZ
OH_t^M	3570	3570	3559	3614	65	67	59
OH_g^M	3543	3543	3539	3591	68	71	54
NH_g^M	-	3396	3396	3438	3	3.8	93
NH_t^M	-	3340	3351	3389	0.4	0.8	76

Attempts to relax the monomers between trans (t) and gauche (g) conformation are described in Fig. S26. For neon as the carrier gas, increasing the stagnation pressure does not lead to a significant change in the t/g ratio, which implies a very slight t advantage. Replacement of Ne by N_2 does increase the relative g intensity somewhat, but this is accompanied by cluster formation and might reflect a more pronounced tendency of t to aggregate or also the onset of N_2 condensation of the monomers.

According to harmonic calculations (Table S22), trans (t) is probably slightly more stable than gauche (g). While this prediction is fairly robust across the different theory levels and also extends to the thermally excited stagnation conditions (Gibbs free energy), it is subtle enough to change with anharmonic corrections.

Therefore, experiment and theory show no clear preference for either trans or gauche as the global minimum structure and the interconversion barrier is too high for an unambiguous experimental clarification.

Table S22: Relative electronic energy ΔE_{el}, harmonically zero point corrected energy ΔE_0 and harmonically estimated standard Gibbs free energy ΔG_0° at 298.15 K (all in kJ mol^{-1}) for the gauche (g) conformer of methylaminoethanol (MAE) relative to the t(rans) conformer using different levels of computation.

Method	ΔE_{el}/(kJ/mol)	ΔE_0/(kJ/mol)	ΔG_0°/(kJ/mol)
B3LYP/maTZ	0.19	0.61	0.78
DLPNO-CCSD(T) B3LYP/maTZ	0.35	0.77	
B2PLYP/QZ	0.18	0.63	0.82
DLPNO-CCSD(T) B2PLYP/QZ	0.32	0.77	

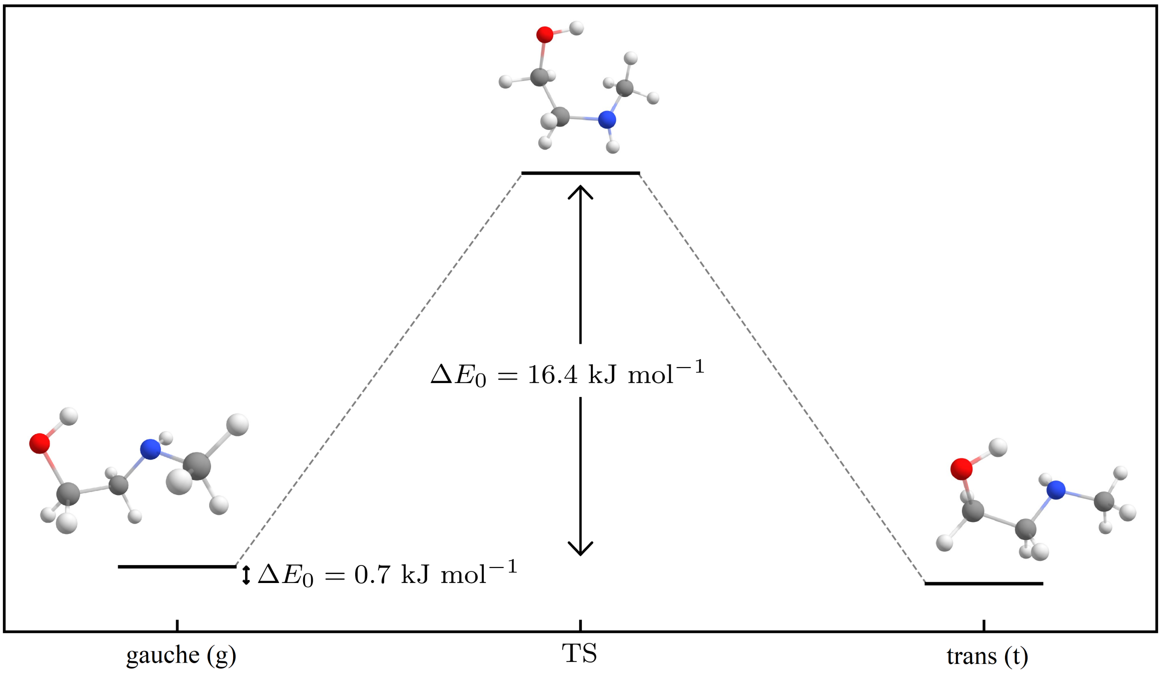

Figure S25: The energy barrier between the most stable gauche (g) conformer and the global minimum trans (t) conformer of MAE The structures shown are optimized at B3LYP-D3/TZ level and ΔE_0/(kJ/mol) is the relative zero point corrected energy.

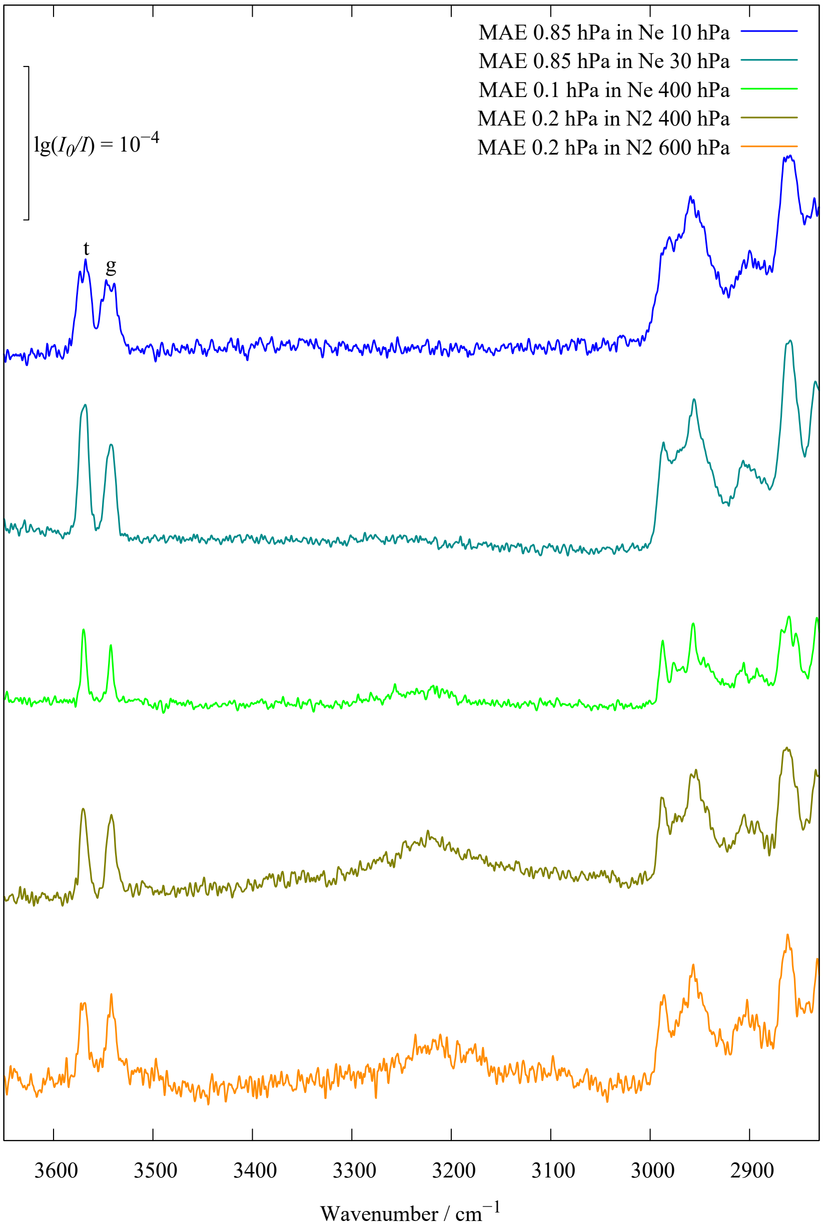

Figure S26: IR spectra of methylaminoethanol (MAE) for different expansion conditions with Ne (top) and N_2 (bottom) as carrier gases. The intensity of trans (t) is higher than gauche (g) with Ne and the intensity ratio of trans (t) and gauche (g) is slightly changed when N_2 is used as carrier gas.

5.5 Dimethylaminopropanol (DMAP)

Raman and FTIR spectra of DMAP are shown in figure S27.

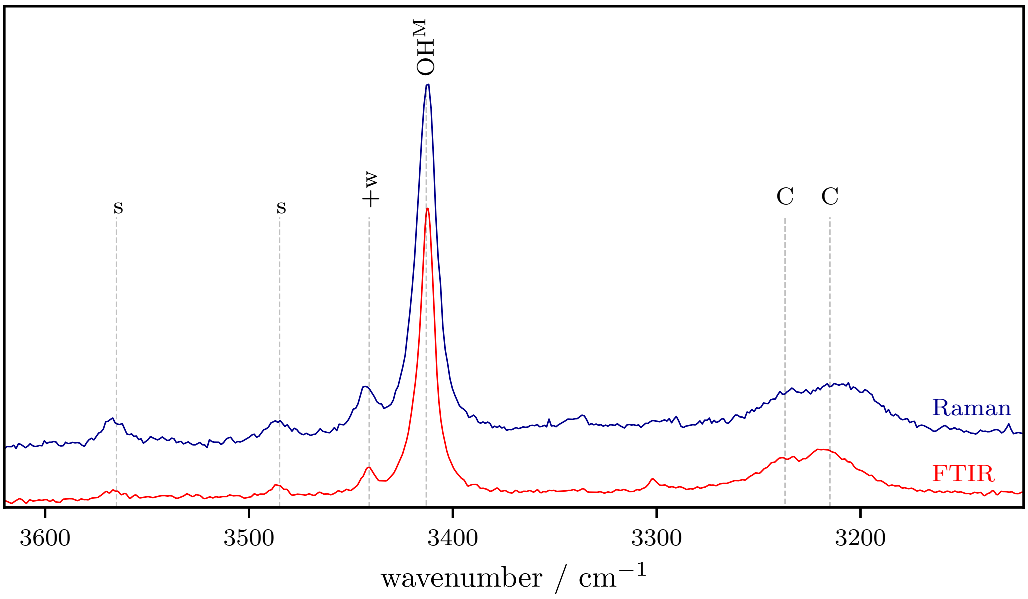

Figure S27: Raman and FTIR spectra of DMAP at cryogenic, supersonic expansion conditions. Sum bands are labelled "s", bands arising from structures including water are labelled "+w", and unknown bands likely arising from DMAP clusters are labelled "C".

There are no clear assignments regarding the cluster bands for DMAP, labelled "C" in Fig. S27 so far.

Table S23: Overview of the results for B3LYP-D3 calculations using the maTZ basis for DMAP. The table lists the relative electronic energy to the most stable conformer ΔE_{el}, the relative ZPVE-corrected energy ΔE_0, the harmonic OH-stretching frequency ω_{OH}, the IR-intensity I_{IR}, the Raman intensity I_{Raman} and the experimental wavenumbers $\tilde{\nu}$ for both FTIR and Raman experiments.

	Theory					Experiment	
conformer	ΔE_{el} / kJ mol^{-1}	ΔE_0 / kJ mol^{-1}	ω_{OH} / cm^{-1}	I_{IR} / km mol^{-1}	I_{Raman} / Å^4 u^{-1}	$\tilde{\nu}_{FTIR}$ / cm^{-1}	$\tilde{\nu}_{Raman}$ / cm^{-1}
Monomer							
M	-	-	3554	366	112	3413	3413

5.6 Comparison of investigated compounds

FTIR spectra of all amino alcohols studied in this work are shown in Fig. S28. One can see the coincidental match of the monomer transitions of the two MAE conformations with those of the single DMAE and AE conformations. One can also see the large differences in dimerisation features - simplicity for AE, overlapping transitions for MAE, clearly separated quartet of isomers for DMAE and overlapping transitions for DMAP.

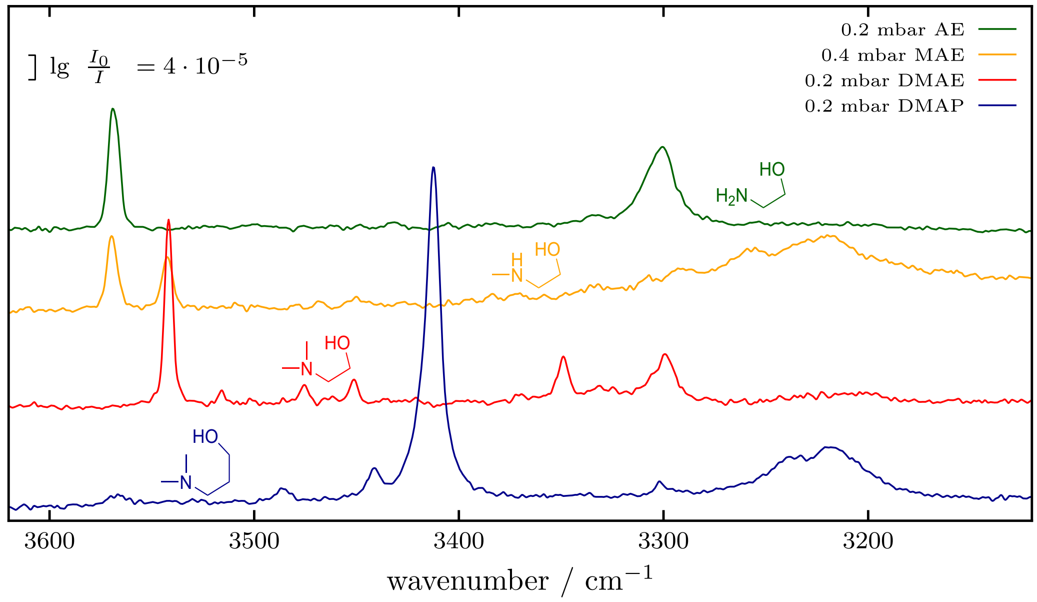

Figure S28: FTIR spectra of all investigated amino alcohols. All compounds were measured in 400 hPa of neon.

6 Entries for the HyDRA database

Comparisons between theory and experiment for Fig. 9 and 10 from the main text are given in Table S24.

Table S24: Experimentally observed (assigned and unassigned) wavenumbers $\tilde{\nu}$ compared to harmonic B3LYP/maTZ predictions ω unscaled and scaled by 0.96 for the monohydrate of dimethylaminoethanol (DMAE) and spectral intensities for Fig. 9 and 10 in the main document. The experimental wavenumbers of DMAE monomer and dimers are also shown.

Species	$\tilde{\nu}$/cm^{-1}	ω/cm^{-1}	0.96 ω/cm^{-1}	I_{IR} / km mol^{-1}	I_{Raman} / Å^4 u^{-1}
i(DMAE+H_2O)					
**OHb$_w$**	3109	3216	3087	947	212
**OH$_A$**	3517	3591	3447	453	72
DMAE+H_2O					
	3503				
	3464				
DMAE monomer					
M	3542	3690	3542		
DMAE dimer					
D	3476	3572	3429		
D	3451	3556	3414		
D	3349	3453	3315		
D	3299	3411	3275		

Table S25: Suggested water OHb stretching wavenumber entry (OHb-r = OHb-d in the absence of an assigned perturbation) for the monohydrate of DMAE in cm^{-1} and expected minimal purity P for the HyDRA database derived from this work. The DMAE monohydrate transition is broader and is thus assigned a larger uncertainty than in amine monohydrates.

Amino alcohol	OHb-r[a]	P	OHb-d[b]
DMAE	3109(12)	≥ 0.90	3109(12)

[a] **r**aw monohydrate wavenumber
[b] **d**eperturbed monohydrate wavenumber

References

[1] Sigma-Aldrich, Ethanolamine safety data sheet, https://www.sigmaaldrich.com/DE/en/sds/sial/e9508?userType=anonymous, accessed: 2024-07-22.

[2] Sigma-Aldrich, 2-(Methylamino)ethanol safety data sheet, https://www.sigmaaldrich.com/DE/en/sds/aldrich/471445?userType=anonymous, accessed: 2024-07-22.

[3] Sigma-Aldrich, Dimethylaminoethanol safety data sheet, https://www.sigmaaldrich.com/DE/en/sds/aldrich/471453?userType=anonymous, accessed: 2024-07-22.

[4] Sigma-Aldrich, Dimethylaminopropanol safety data sheet, https://www.sigmaaldrich.com/DE/en/sds/aldrich/d144401?userType=anonymous, accessed: 2024-07-22.

[5] H. C. Gottschalk, T. L. Fischer, V. Meyer, R. Hildebrandt, U. Schmitt, M. A. Suhm, *Instruments* **2021**, *5*, 12.

[6] N. O. B. Lüttschwager, *Phys. Chem. Chem. Phys.* **2024**, *26*, 10120–10135.

[7] G. Herzberg, *Molecular Spectra and Molecular Structure - Vol I*, D. Van Nostrand company, **1950**.

[8] N. O. B. Lüttschwager, *J. Open Source Softw.* **2021**, *6*, 3526.

[9] S. Grimme, J. Antony, S. Ehrlich, H. Krieg, *J. Chem. Phys.* **2010**, *132*, 154104.

[10] S. Grimme, S. Ehrlich, L. Goerigk, *J. Comput. Chem.* **2011**, *32*, 1456–1465.

[11] F. Weigend, R. Ahlrichs, *Phys. Chem. Chem. Phys.* **2005**, *7*, 3297–3305.

[12] W. Sander, S. Roy, I. Polyak, J. M. Ramirez-Anguita, E. Sanchez-Garcia, *J. Am. Chem. Soc.* **2012**, *134*, 8222–8230.

[13] D. Leicht, M. Kaufmann, R. Schwan, J. Schäfer, G. Schwaab, M. Havenith, *J. Chem. Phys.* **2016**, *145*, 204305.

[14] F. Neese, *Wiley Interdiscip. Rev. Comput. Mol. Sci.* **2012**, *2*, 73–78.

[15] F. Neese, *Wiley Interdiscip. Rev. Comput. Mol. Sci.* **2022**, *12*, e1606.

[16] M. A. Marques, M. J. Oliveira, T. Burnus, *Comput. Phys. Commun.* **2012**, *183*, 2272–2281.

[17] S. Lehtola, C. Steigemann, M. J. Oliveira, M. A. Marques, *SoftwareX* **2018**, *7*, 1–5.

[18] S. Grimme, *J. Chem. Theory Comput.* **2019**, *15*, 2847–2862.

[19] P. Pracht, F. Bohle, S. Grimme, *Phys. Chem. Chem. Phys.* **2020**, *22*, 7169–7192.

[20] S. Grimme, *J. Chem. Phys.* **2006**, *124*, 034108.

[21] C. Riplinger, B. Sandhoefer, A. Hansen, F. Neese, *J. Chem. Phys.* **2013**, *139*, 134101.

[22] R. A. Kendall, J. Dunning, Thom H., R. J. Harrison, *J. Chem. Phys.* **1992**, *96*, 6796–6806.

[23] D. E. Woon, J. Dunning, Thom H., *J. Chem. Phys.* **1993**, *98*, 1358–1371.

[24] A. K. Wilson, D. E. Woon, K. A. Peterson, J. Dunning, Thom H., *J. Chem. Phys.* **1999**, *110*, 7667–7676.

[25] V. Ásgeirsson, B. O. Birgisson, R. Bjornsson, U. Becker, F. Neese, C. Riplinger, H. Jónsson, *J. Chem. Theory Comput.* **2021**, *17*, PMID: 34275279, 4929–4945.

[26] B. Dmytro, P. Taras, I. Róbert, K. Simone, B. Ute, V. Edward, N. Frank, *Mol. Phys.* **2015**, *113*, 1961–1977.

[27] P. Asselin, B. Madebène, P. Soulard, R. Georges, M. Goubet, T. R. Huet, O. Pirali, A. Zehnacker-Rentien, *J. Chem. Phys.* **2016**, *145*, 224313.

[28] S. H. Yazdabadi, D. Mihrin, K. L. Feilberg, R. W. Larsen, *J. Chem. Phys.* **2024**, *161*, 154301.

[29] Y. Liu, C. A. Rice, M. A. Suhm, *Can. J. Chem.* **2004**, *82*, 1006–1012.

[30] M. Gawrilow, M. A. Suhm, *Phys. Chem. Chem. Phys.* **2020**, *22*, 15303–15311.
